# Host Invasion by Pathogenic Amoebae: Epithelial Disruption by Parasite Proteins

**DOI:** 10.3390/genes10080618

**Published:** 2019-08-14

**Authors:** Abigail Betanzos, Cecilia Bañuelos, Esther Orozco

**Affiliations:** 1 Consejo Nacional de Ciencia y Tecnología (CONACYT), Mexico City 03940, Mexico; 2 Departamento de Infectómica y Patogénesis Molecular, Centro de Investigación y de Estudios Avanzados del Instituto Politécnico Nacional (CINVESTAV-IPN), Mexico City 07360, Mexico; 3 Coordinación General de Programas de Posgrado Multidisciplinarios, Programa de Doctorado Transdisciplinario en Desarrollo Científico y Tecnológico para la Sociedad, CINVESTAV-IPN, Mexico City 07360, Mexico; 4 Departamento de Infectómica y Patogénesis Molecular, CINVESTAV-IPN, Mexico City 07360, Mexico

**Keywords:** epithelia, tight junctions, adherens junctions, desmosomes, *Entamoeba histolytica*, *Naegleria fowleri*, *Acantamoeba* spp.

## Abstract

The epithelium represents the first and most extensive line of defence against pathogens, toxins and pollutant agents in humans. In general, pathogens have developed strategies to overcome this barrier and use it as an entrance to the organism. *Entamoeba histolytica*, *Naegleria fowleri* and *Acanthamoeba* spp. are amoebae mainly responsible for intestinal dysentery, meningoencephalitis and keratitis, respectively. These amoebae cause significant morbidity and mortality rates. Thus, the identification, characterization and validation of molecules participating in host-parasite interactions can provide attractive targets to timely intervene disease progress. In this work, we present a compendium of the parasite adhesins, lectins, proteases, hydrolases, kinases, and others, that participate in key pathogenic events. Special focus is made for the analysis of assorted molecules and mechanisms involved in the interaction of the parasites with epithelial surface receptors, changes in epithelial junctional markers, implications on the barrier function, among others. This review allows the assessment of initial host-pathogen interaction, to correlate it to the potential of parasite invasion.

## 1. Introduction

About 15 million annual human deaths worldwide are directly related to infectious diseases [1]. Neglected diseases and healthcare-associated infections, as well as new and emerging pathogens, are increasing challenges [2]. Therefore, further research should address gaps existing in the knowledge of parasites biology, host-parasite interactions, mechanisms of pathogenesis, evasion of the host immune response, and development of parasites drug-resistance.

The entrance of pathogens to the host, typically occurs through natural cavities such as the mouth, eyes, nose, or genital openings, or by wounds that disrupt the skin barrier, overall covered or lined by epithelia [2].

**Figure 1:**
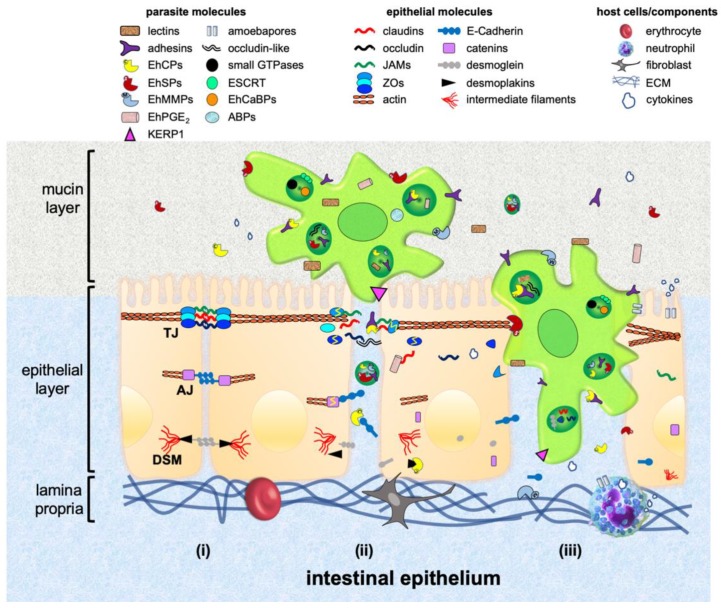
Model of invasion of the intestinal epithelium by *E. histolytica*. (**i**) Representative scheme of the intestinal epithelium as a selective barrier and intercellular junctions (IJs) (tight junctions (TJs), adherens junctions (AJs) and desmosomes (DSMs)), as well as the extracellular matrix (ECM). (**ii**) At the beginning of contact, trophozoites penetrate the mucin layer by employing adhesins and lectins for attaching, and proteases for degrading mucin. The parasite attaches to epithelial cells, particularly at the intercellular space, opening IJs with the participation of adhesins, proteases, prostaglandin and occludin-like proteins. IJs components are selectively degraded, displaced by competition and internalized by trophozoites. (**iii**) Once the barrier is disrupted, epithelial cells are separated and lysed, allowing the entrance of trophozoites toward the lamina propria. In this stage, the participation of endosomal sorting complex required for transport (ESCRT) machinery components, calcium binding-proteins (EhCaBPs), small GTPases, actin binding-proteins (ABPs), *E. histolytica* metalloproteases (EhMMPs) and amoebapores, is crucial. In response, the host immune system recruits neutrophils and induces cytokines production. EhCPs: *E. histolytica* cysteine proteases; EhSPs: *E. histolytica* serine proteases; EhPGE_2_: prostaglandin E_2_; ZOs: *zonula occludens* proteins.

Epithelia are formed by tightly cohesive sheets of cells, which cover or line body surfaces, such as skin, gut, nose and others, and also function as secretory glands, like salivary tissue and the pancreas. Epithelial functions are largely due to the arrangement of cells, firmly joined together via adhesive structures, anchoring the cytoskeleton of each cell to its neighbours, and to underlying or surrounding extracellular matrix (ECM) components [3]. These adhesive structures, known as intercellular junctions (IJs), are mainly organized in tight junctions (TJs), adherens junctions (AJs) and desmosomes (DSMs), and localize at the lateral cell membrane [4] (Figure 1).

Tight junctions are the most apico-lateral cell-cell contact and represent the first defiance for pathogens penetrating the host epithelium (Figure 1). They are composed of transmembrane (TM) proteins, such as occludin, claudins and junctional adhesion molecules (JAMs), which form dimmers with their equivalent proteins from neighbouring cells; and by the cytoplasmic plaque, formed by *zonula occludens* proteins (ZOs), cingulin, membrane-associated guanylate kinases with inverted domains structure and partitioning-defective proteins (PARs), which bind to the actin cytoskeleton [5]. Tight junctions regulate the flux of macromolecules and ions through the paracellular route, and also maintain the polarization of lipids and proteins in the plasma membrane [6]. Behind TJs, AJs and DSMs are located. They give strength to the epithelium and preserve it as a strong and selective fence against pathogens and harmful compounds [7,8]. Adherens junctions maintain TJs integrity and tissue homeostasis [9]. Some AJs proteins (afadin, nectin and β-catenin) participate in intracellular signalling and transcriptional regulation. Others, such as the TM glycoproteins of the cadherin superfamily (E-cadherin), have cytoplasmic tails that bind to catenins, which interact with the actin cytoskeleton, giving full adhesive activity to the cells [7]. Desmosomes maintain the epithelium integrity by strong extracellular bonds fastened to the intermediate filaments [8]. They are also constituted by cadherins (desmoglein and desmocollin) that establish the adhesive interface of these structures, and interact with plaque proteins (desmoplakin, plakophilin and plakoglobin) to allow contact with intermediate filament-linking proteins [8,10].

The intestinal epithelium is covered by the mucous layer containing mucin with antimicrobial properties. Underlying this stratum, there is a single layer of absorptive epithelial, goblet and Paneth cells that function as a barrier, selectively allowing the passage of nutrients and molecules (Figure 1). In counterpart, it avoids the ingress of antigens, toxins and pathogens to the human body. Amino acids, electrolytes, sugars and some proteins cross over the transcellular route by endocytosis, using specific molecules located at the cell apical surface. Other molecules, as well as pathogens and cells, also cross through the paracellular route using the space between two or more epithelial cells. The epithelial layer also maintains a healthy relationship between gut microbiota and host immunity. Immune cells coexist with ECM components at the lamina propria, which lies under epithelial cells [11].

**Figure 2:**
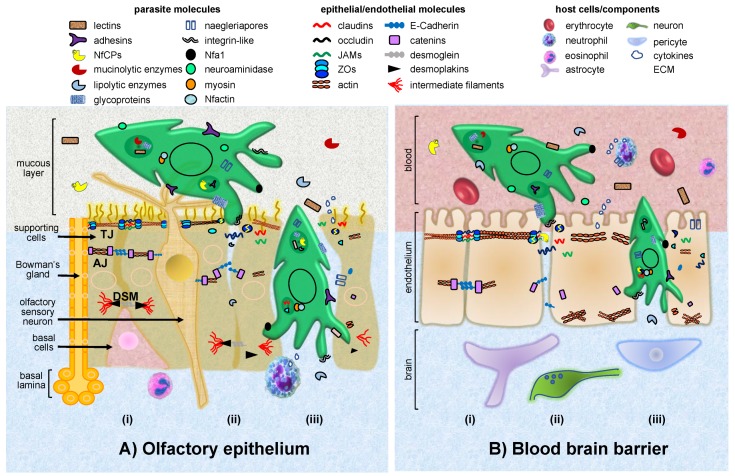
Model of invasion of the olfactory epithelium and blood brain barrier by *N. fowleri.* (**A**) Representative scheme of the pseudostratified olfactory epithelium invasion by trophozoites. (**i**) TJs fasten the supporting cells, Bowman’s glands and olfactory sensory neurons. Cell binding among them and basal cells is reinforced by AJs and DSMs. (**ii**) At the beginning of contact, trophozoites penetrate the mucous layer employing adhesins, lectins, CPs and mucinolytic enzymes. The parasite attaches to epithelial cells, particularly at the intercellular space, opening IJs with the participation of adhesins, glycoproteins, proteases and integrin-like proteins. IJs components are selectively degraded, displaced by competition and internalized by trophozoites. (**iii**) Once the barrier is disrupted, epithelial cells are separated and lysed, allowing the entrance of trophozoites toward the basal lamina, to eventually invade the CNS. In this stage, the participation of lipolytic enzymes, naegleriapores, *N. fowleri* antigen-related protein 1 (Nfa1), myosin and *N. fowleri* actin (Nfactin), is crucial. (**B**) Representative scheme of the blood brain barrier invasion by trophozoites. (**i**) Endothelial cells are joined by TJs and AJs. (**ii**) Trophozoites coming from the blood stream adhere to endothelial cells, particularly at the intercellular space, opening IJs. (**iii**) Once the barrier is disrupted, trophozoites gain access to neurons and glia cells. The parasite molecules involved in epithelial damage are the same that participate in endothelial invasion. In response, the host immune system recruits neutrophils and eosinophils, and induces cytokines production.

The olfactory epithelium is composed of several cell types, including basal cells, olfactory sensory neurons and supporting cells (Figure 2). Basal cells, resting on or near the basal lamina, are stem cells that remain relatively quiescent until they differentiate into either supporting or olfactory cells. Olfactory sensory neurons are bipolar neurons that sense environmental chemicals and maintain the ability to regenerate throughout adulthood. Supporting cells located at the apical layer of the pseudostratified ciliated columnar epithelium, act as metabolic and physical basement for the olfactory epithelium, and also detoxify potentially dangerous chemical agents [12,13].

**Figure 3:**
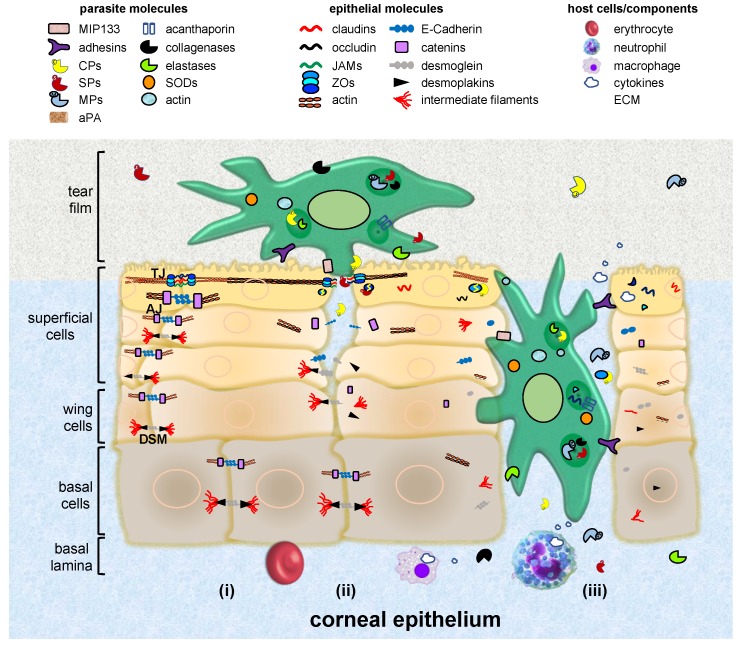
Model of invasion of the corneal epithelium by *Acanthamoeba* spp. (**i**) Representative scheme of the corneal stratified epithelium as an efficient barrier due to TJs displayed at the upmost layer of superficial cells. The next layers, as well as the wing and basal cells, only exhibit AJs and DSMs. (**ii**) Trophozoites adhere to epithelial cells, particularly at the intercellular space, opening IJs with the participation of adhesins, proteases and cytolytic mannose-induced protein of 133 kDa (MIP133). Consequently, IJs components are selectively degraded and internalized. (**iii**) Once the barrier is disrupted, epithelial cells are separated and lysed, allowing the entrance of trophozoites toward the basal lamina and eventually reaching the CNS. In this stage, the participation of acanthaporin, proteases, actin and superoxide dismutases (SODs), is crucial. In response, the host immune system recruits neutrophils and macrophages, and induces cytokines production.

The corneal epithelium is a non-keratinized, highly-organized, non-secretory squamous and stratified tissue, with five to seven cell layers’ depth (Figure 3). These layers are constituted by three cell types: superficial, wing and basal cells [14]. The first ones are terminally differentiated cells with apical surface projections (microvilli) that express an adherent glycocalyx to anchor to the tear film. Eventually, these cells degenerate and slough from the surface. Wing cells are partially differentiated and adopt a wing shape phenotype. Basal cells form a single layer of cuboidal cells adhered to a basement membrane with mitotic activity [15].

Breakage of epithelial barriers by microorganisms’ action is the first step for invasion [11,16,17,18]. Some viruses, bacteria and protozoa directly use proteins of the host IJs to penetrate the epithelium, triggering inflammatory processes that involve immune cells action [19]. In response, pathogens produce toxic molecules and proteases that alter IJs, cytoskeleton and other cell structures to reach and invade additional tissues [20]. Viruses use the adhesion molecules of the host as receptors, such as the ανβ1- and ανβ3-integrins, recognized by the major capsid protein of human parechovirus 1 [21]; the intercellular adhesion molecule 1 (ICAM1), which is used as a receptor by human rhinoviruses [22]; the decay-accelerating apical protein (DAF), widely employed by enteroviruses [23]; and IJs proteins, as claudins and occludin, both exploited by the core protein 8 of rotavirus to provoke the disruption of the junctional complex, compromising the epithelial barrier integrity [24]. Bacteria, such as *Helicobacter pylori*, provoking gastritis, gastroduodenal ulcers and gastric cancer, attaches close to IJs and delivers the cytotoxin-associated gene A product via the type IV secretory system into the epithelial cytosol [25]. Subsequently, the bacterium induces the remodelling of cell-cell junctions, which is executed through the activation of the myosin light-chain kinase (MLCK)/myosin light-chain (MLC) signalling pathway and the mislocalization of ZO-1 and JAM proteins [26].

Protozoa use their membrane proteins or secreted molecules to interact with epithelial receptors [16,17,27]. The most important pathogenic amoeba for humans are *Entamoeba histolytica*, *Naegleria fowleri* and *Acanthamoeba* spp. [28,29,30]. *E. histolytica* causes human amoebiasis and produces up to 100,000 deaths worldwide each year [31]. It is transmitted by the ingestion of water or food contaminated with faeces or via oral-anal sexual practices. After ingestion of cysts, excystment occurs in the small intestine and trophozoites move by pseudopodia, attaching to the intestinal epithelium and disrupting IJs to penetrate it [29,32]. By the bloodstream, the parasite may reach other organs, including the brain and the liver [33]. Diarrhoea is the most common manifestation of the disease, followed by dysentery and occasionally, extra-intestinal abscesses, mainly in liver [34].

*N. fowleri*, colloquially known as the “brain-eating amoeba”, is a free-living protozoa, responsible for primary amoebic meningoencephalitis (PAM) in humans [35]. This disease produces 90 to 95% lethality rates, after just three to seven days of parasite infection [36]. *N. fowleri* presents amoeboid and cyst forms, besides a flagellate intermediate stage, which are extensively dispersed in the environment and able to produce infection [37,38]. This amoeba accesses the host during swimming or diving, by attaching to human olfactory epithelium and penetrating mucous membranes from the nasal route, to finally reach the central nervous system (CNS) [28]. Once in the brain, the amoebae cause extensive tissue damage and inflammation, lysing and ingesting erythrocytes and other cell types, such as nerve cells. The destruction of tissue and haemorrhagic necrosis of the brain is accompanied by an inflammatory infiltrate that consists of neutrophils, eosinophils, and macrophages [39].

*Acanthamoeba* spp. are opportunistic free-living amoebae that cause amoebic encephalitis (GAE), amoebic keratitis (AK), pneumonitis, or changes in other organs, such as liver, kidneys and skin, particularly in immunocompromised individuals [30,40]. Based on morphological criteria, as many as 24 species have been included in the genus *Acanthamoeba*. However, only some of them result pathogenic to humans, including *A. castellanii*, *A. culbertsoni*, *A. polyphaga*, *A. rhysodes*, *A. divionensis*, *A. hatchetti*, *A. healyi*, and *A. lenticulate* [41]. Recently, an increasing number of *Acanthamoeba* infections has been observed worldwide, reporting values from 17 to 70 AK cases per million of contact lens wearers [42]; while, more than 400 cases of GAE have been reported, with only a 2 to 3% patient survival rate [43]. *Acanthamoeba* spp. present two stages during their life cycle, the infective trophozoite and the dormant cyst, which may also serve as reservoirs for bacteria and virus with pathogenic potential for humans [41]. The route of amoebae infection is through corneal abrasions, olfactory epithelium, respiratory tract, skin or sinuses, to cross the blood-brain barrier and enters into the CNS. Pathological manifestations of AK include ulceration of cornea, whereas in GAE focal necrosis and granulomas are observed [44].

In this work, we reviewed the major proteins and events from *E. histolytica*, *N. fowleri* and *Acanthamoeba* spp. related to host interactions, with special emphasis in those that affect IJs to invade epithelia. A better understanding of the biology of parasite proteins and molecules relevant for host invasion, may contribute to the development of efficient therapeutic drugs against pathogenic amoebae.

## 2. *Entamoeba histolytica*

The destructive mechanisms of *E. histolytica* encompass contact with target cells, cytolysis, phagocytosis and degradation of ingested cells. After contact with trophozoites, the epithelial cells increase the paracellular permeability produced by TJs opening and microvilli distortion. Surface blebbing and minute focal discontinuities in the host plasma membrane appear, causing the displacement and separation of neighbouring cells, and compromising the membrane integrity [45,46,47] (Figure 1 and Table 1).

### 2.1. Penetration of Mucous Layer

To trespass the mucous barrier present in the intestinal epithelium, *E. histolytica* binds to mucin through the d-galactose and *N*-acetyl-d-galactosamine (EhGal/GalNAc) lectin, cysteine proteases (EhCPs) and glycosidases [48,49,50].

The EhGal/GalNAc lectin is a 260 kDa heterotrimeric complex composed by the heavy (Hgl, 170 kDa), intermediate (150 kDa) and light (35/31 kDa) subunits [51]. The Hgl subunit displays the carbohydrate recognition domain (CRD) [52] that interacts with the abundant galactose and *N*-acetyl-galactosamine residues present in mucin [48].

*E. histolytica* cysteine proteases (EhCPs) exert the major mucin degradation. Even though *E. histolytica* possesses over 50 EhCPs [53], only two of them, EhCP112 and EhCP5, degrade mucin [54,55].

EhCP112 couples with the EhADH adhesin to form the EhCPADH complex, which is involved in several virulence mechanisms [56,57]. The EhCPADH complex, as well as EhADH and EhCP112, are peripheral membrane proteins, also localized in cytoplasmic vesicles, susceptible of being secreted by trophozoites [54,58,59]. EhCP112 harbours a canonical l-cathepsin catalytic domain (Cys-His-Asn) and an Arg-Gly-Asp residues (RGD) sequence that in other organisms interacts with host integrins [57,60]. This protein was localized in the mucous layer of mice, luminally inoculated with a recombinant EhCP112 polypeptide. In addition, immunohistochemical assays evidence that EhCP112 and mucin-2 colocalize in the colon, strongly suggesting that this enzyme accesses the colonic epithelial cells by degrading mucin. In response, the host evokes mucous hypersecretion to repel the parasite invasion, as evidenced in mice treated with EhCP112, which exhibit an increase of mucin-2 in colon tissues [54]. EhCP112 presents proteolytic activity on gelatine, collagen type I, fibronectin, haemoglobin and on Z-Phe-Arg, a specific substrate for l-cathepsins [54,58,60].

EhCP5 is present on the surface of trophozoites and is also secreted [61]. In *ehcp5*-silenced trophozoites, mucin degradation is significantly diminished (>60%), compared to wild-type trophozoites. Similarly, recombinant EhCP5 degrades >45% of purified native mucin, and the CPs inhibitor, E64, specifically blocks the EhCP5 proteolytic action [62]. Besides, EhCP5 couples to αvβ3 integrin on goblet cells, inducing mucin hypersecretion [55].

In addition, *E. histolytica* contains several glycosidases localized at the parasite membrane, such as sialidase, *N*-acetyl-galactosamidase and *N*-acetyl-glucosaminidase. Some of them remove branched polysaccharides from mucin, contributing to the mucous layer degradation [50].

### 2.2. Adherence of Trophozoites to the Host Epithelium

Trophozoites adhere to host cells mainly by adhesins and lectins [63]. The most well-known molecules are the EhCPADH complex and the EhGal/GalNAc lectin [51,56]. However, other molecules seem to be involved in this episode, such as the 220-lectin [64,65], a lysine and glutamic acid enriched protein (KERP1) [66], the pyruvate:ferredoxin oxidoreductase (PFO) [67] and the *E. histolytica* rhomboid protein 1 (EhROM1) [68].

Monoclonal antibodies against the EhCPADH complex diminish the ability of parasites to make contact with epithelial cells and erythrocytes [56]. EhADH and EhCP112 also contribute to the adherence to target cells. EhADH contains a Bro1 domain at its amino terminus, characteristic of ALG-2 interacting protein-X (ALIX) family members, and a carboxy terminus adherence epitope, which is recognized by monoclonal antibodies that inhibit adherence [56,69]. Madin-Darby canine kidney cells (MDCK) cells ectopically expressing EhADH, exhibit epithelial aggregation and adherence to erythrocytes, due to the properties conferred by the parasite adhesin [70]. Trophozoites silenced in the *ehadh* or *ehcp112* genes show 65% and 75% reduction of adherence to host cells, respectively [71]. In addition, recombinant EhADH and EhCP112 proteins bind to epithelial cells and erythrocytes [54,70,72]. 

The EhGal/GalNAc lectin interacts with glycoproteins present on the host cell membrane through its CRD motif [51]. Interestingly, the CRD motif presents sequence similarity with regions from the cytoplasmic tail of host β2 integrin [73], and a calcium-binding motif [52] comparable to several calcium-dependent epithelial-cadherins [74]. Antibodies directed against the Hgl subunit diminish the ability of trophozoites to make contact with epithelial cells [75]. The EhGal/GalNAc lectin also binds to ECM components, and erythrocytes and leukocytes, which lies beneath the epithelium [48,76]. All this data, along with a transcriptomic analysis of axenically cultured *E. histolytica* trophozoites, confirmed the participation of this lectin in adherence and other virulence events [77].

A 220-lectin, rich in hydrophobic residues, binds to hyaluronic acid, chitin, chitotriose, *N*-acetyl-galactosamine and galactose [64], and has been also involved in trophozoites adherence to MDCK cells and erythrocytes [64,65].

EhROM1 is an intramembrane serine protease (SP), which cleaves transmembrane proteins in, or in close proximity to, their transmembrane domain. Trophozoites *ehrom1*-epigenetically silenced, diminish their adherence to Chinese hamster ovary cells (CHO) epithelial cells [68]. In addition, EhROM1 cleaves the Hgl subunit of the EhGal/GalNAc lectin, suggesting a delicate regulation of lectins by *E. histolytica* serine proteases (EhSPs) during parasite adherence to host cells [78].

KERP1 harbours three coiled-coil regions within its central segment, contributing to trimer formation and adherence to human colorectal adenocarcinoma cells (Caco-2) cells [66,79]. Otherwise, PFO catalyses the oxidative decarboxylation of pyruvate, transferring electrons to ferredoxin. This enzyme produces the cytotoxic activation of metronidazole, the drug most widely used against amoebiasis [80]. Remarkably, KERP1 and PFO are localized at the plasma membrane of trophozoites adhered to Caco-2 cells, close to the intercellular space [79,80].

### 2.3. Interaction with Epithelial Intercellular Junctions

Once *E. histolytica* makes contact with the epithelium, trophozoites pose on the apical part of the intercellular space, where IJs are situated [45]. Next, they produce a dramatical drop of the transepithelial electrical resistance (TEER). TEER dropping is accompanied by an increase of the paracellular permeability, indicating IJs disruption [32,45,81,82]. Trophozoites extracts and secreted proteins produce a similar effect [45,82]. The EhCPADH complex [82], EhCPs [54,83,84], EhSPs [85], prostaglandin E_2_ (EhPGE_2_) [86] and an occludin-like protein [87] participate in this event.

EhCPADH, EhADH and EhCP112 have been detected at TJs, co-localizing with occludin and claudin-1 in epithelial cells incubated with trophozoites [54,70,82]. Moreover, EhCPADH and EhCP112 are involved in the TEER dropping, in a time-dependent manner. Accordingly, degradation of claudin-1, occludin, ZO-1 and ZO-2 occurs [54,82]. Conversely, at early times of contact with trophozoites or EhCP112, claudin-2 appears in a higher amount in TJs, but later, is degraded [54]. Claudin-2 is abundant in leaky epithelia, allowing the exit of cations [88], thus explaining its sudden abundance as a consequence of the epithelium loosening. Consistently, in the colon of *muc2^−/−^* KO mice infected with *E. histolytica*, claudin-2 expression increases, correlating with a greater paracellular permeability [32].

A comparative study, co-incubating purified recombinant EhCP1, EhCP2, EhCP5 and EhCP112 enzymes with Caco-2 cells, showed that EhCP112 has the most potent activity on TEER [54]. EhCPs also participate in the villin proteolysis [89]. EhCP1, EhCP2 and EhCP4, degrade human villin, and destroy the microvilli integrity [84,85,90]. Furthermore, EhCP1, EhCP4 and EhCP6 are upregulated in trophozoites isolated from mice colon, suggesting a participation during invasion and colonization of epithelium [91]. Accordingly, trophozoites with low levels of EhCPs activity are incapable of damaging the intestinal barrier in human colonic xenografts [92]. Otherwise, in Caco-2 cells incubated with trophozoites pre-treated with tosyl-l-lysyl-chloromethane hydrochloride and tosyl phenylalanyl chloromethyl ketone (two CPs and SPs inhibitors, respectively), the proteolysis of ZO-1, ZO-2 and villin is prevented. These data suggest that *E. histolytica* overcomes microvilli and TJs barriers by using EhCPs and EhSPs [85,89].

EhPGE_2_ secreted by *E. histolytica*, also produces TEER dropping in a dose- and time-dependent manner, probably due to the dissociation of claudin-4 from TJs. The EhPGE_2_ signal is transduced through the prostaglandin E_2_ receptor 4 (EP_4_) of epithelial cells. The altered paracellular permeability favours the sodium exit towards the lumen, while EhPGE_2_ induces a luminal chloride secretion. Then, the NaCl gradient triggers the osmotic water flow across the epithelium, contributing to diarrhoea [86].

*E. histolytica* expresses an occludin-like protein (55 kDa) with similarities to the extracellular loops of human occludin [87], which interacts in an homotypic manner to create and maintain the epithelial barrier [93]. The occludin-like protein has not been detected in the plasma membrane, but it is involved in the TEER dropping of T84 human colorectal carcinoma cells. Thus, it is possible that this protein displaces host cellular occludin interactions, disrupting the barrier [87].

After TJs are damaged, AJs and DSMs are reached and destroyed by trophozoites. The recombinant EhCP112 degrades E-cadherin and desmoglein-2, destabilizing the bond with their scaffold partners β-catenin and desmoplakin l/II, respectively. The latter two, appear as also degraded and translocated to the nucleus, probably as a compensatory mechanism carried out by the host cell to repair the damage produced by the parasite [72]. In addition, EhCP112 and EhADH are endocytosed by caveolin or clathrin-coated vesicles and liberated inside the host cell [70,72]. Hence, it is possible that IJs may also be damaged by these proteins, from the intracellular side.

### 2.4. Cytolysis

*E. histolytica* exerts an extensive cytolytic ability, as demonstrated by the swell and massive blebbing of the epithelial surface [94]. Parasite molecules participating in cytolysis include EhCPs and a family of pore-forming peptides termed amoebapores [77,95,96].

EhCPs present a wide variety of host substrates including mucin, epithelial and ECM proteins, immunoglobulins and complement [97]. The *ehcp112* or *ehadh* genes silencing in trophozoites produces a significant reduction (55% and 45%, respectively) of the parasite cytopathic effect on epithelial cells [71,98]. Moreover, antibodies against the EhCPADH complex, inhibit the destruction of MDCK and Caco-2 monolayers by trophozoites, and also reduce trophozoites ability to produce liver abscesses in hamsters [56,82,99,100].

EhCP1 cleaves collagen, the C3 complement factor, IgG, pro-IL-18, pro-IL-1β and villin [97,101]. EhCP2 degrades collagen, IgA, IgG, chemokines (CCL2, CCL13 and CXCL8), C3 and pro-IL-18 [90,102]. In addition to mucin, EhCP5 also degrades collagen, IgA, pro-IL-18, haemoglobin, fibrinogen, BSA and human pro-matrix metalloproteinase (MMP) 3 [55,103].

*E. histolytica* amoebapores are released into the intercellular space and inserted into the membrane of target cells, creating ion channels and resembling the cytotoxic mechanism followed by lymphocytes [96]. The amoebapores family comprises three members, A, B and C, found in a 35:10:1 ratio in trophozoites, that induce the lysis of eukaryotic cells, but differ in their kinetics to create ion channels [96,104]. Trophozoites depleted on amoebapore A, still produce inflammation and tissue damage in the severe combined immunodeficient mouse-human intestinal xenograft model of amoebic colitis. However, its expression is required for full virulence in a mouse model of amoebic liver abscess [105].

The epithelium damage produced by cytolytic molecules of trophozoites, eventually results in cell death, mainly by apoptosis [62,106,107], although autophagy has also been reported [82].

### 2.5. Phagocytosis and Trogocytosis

The ability of *E. histolytica* to phagocytose significantly contributes to the host invasion, hence, trophozoites deficient in ingestion are less virulent [108,109]. Trophozoites phagocytose enterocytes, erythrocytes, fibroblasts, lymphocytes, bacteria and other cells [94]. However, several studies have proposed that trophozoites first kill host cells, before phagocytosing them [94,95,110].

The attachment of *E. histolytica* to the host cells potentially triggers the ingestion process, then actin-myosin rearrangements produce changes in cell shape, and finally the phagosomes formation leads to the cargo degradation [94]. Adhesins [56,71], actin binding-proteins (ABPs) [111,112], calcium binding-proteins (EhCaBPs) [113], small GTPases [114] and intracellular trafficking molecules [115,116,117], among others, participate in this complex event.

EhCPADH is localized in the phagocytic cups of trophozoites during erythrophagocytosis [59,118]. Trophozoites pre-treated with a specific antibody against this complex diminish their phagocytosis rates by 41%, and erythrocytes pre-treated with a polypeptide containing the last 240 amino acids of EhADH are 79% less phagocytosed by trophozoites than control erythrocytes [56]. Moreover, trophozoites with the *ehadh* or *ehcp112* genes silenced, display a reduction in their erythrophagocytosis efficiency of 30% or 60%, respectively [71].

Likewise, the inhibition or decreased expression of EhGal/GalNAc [110,119], EhROM1 [68], some transmembrane kinase family proteins (EhTMK39, EhTMKB1-9 and *E. histolytica* phagosome-associated TMK96 protein (EhPATMK)) [120], exoribonuclease EhRrp6 [121], and the *E. histolytica* metallosurface protease 1 (EhMSP-1) [122], significantly reduces the trophozoites ability to phagocytose.

During phagocytic cup formation, engulfing and phagosome formation, actin undergoes rearrangements, modulated by ABPs as actin-related protein 2/3 complex subunit 1 (ARPC1) [111], 166 kDa nucleocytoplasmic actin-binding protein (NCABP166) [123], and myosin IB [112]. Furthermore, EhCaBPs also participates in the actin remodelling during membrane deformation [113]. In fact, the interaction of EhCaBP3 with EhARPC2 contributes to the recruitment of myosin 1B to the phagocytic machinery, promoting the closure of cups and formation of phagosomes [124]. Additional actin regulators contributing to phagocytosis are small GTPases [125], which also participate in other endocytosis processes, and during invasion [114,125]. *E. histolytica* ras-associated binding protein (EhRab)A interacts with calreticulin and inhibit erythrophagocytosis [126]. EhRabB localizes to the phagocytic cup during phagocytosis [127]. Overexpression of the *ehrab5* gene provokes in trophozoites an increase of their ability to phagocytose and an augment of the transport of amoebapore to phagosomes [128]. EhRab5 together with EhRab7A, localize in unique vacuoles, which are essential for erythrocytes engulfment and for lysosomal hydrolases packaging, previous fusion with phagosomes [128]. EhRab7B plays a role in late endosome-lysosome fusion [129]. The knockdown of the *ehrab8A* gene, diminishes phagocytosis of erythrocytes, bacteria and carboxylated latex beads [130]. EhRab11B is associated with non-acidified vesicles considered as recycling compartments, and regulates the secretion of EhCP1, EhCP2 and EhCP5 [131]. Interestingly, the Hgl subunit of Gal/GalNAc is associated with EhRab5, EhRab7A, and EhRab11B in the early stage of endocytosis, but only EhRab7A remains associated to the Hgl subunit during its transport to lysosomes [132]. The p21racA participation in phagocytosis of mucin-coated beads, bacteria and erythrocytes was demonstrated in trophozoites overexpressing this protein mutated in its GTPase activity to remain constantly in the active state [133]. EhRho1 modulates phagocytosis recruiting EhFormin1 and EhProfilin1, so promoting actin polymerization [134].

In addition, *E. histolytica* possesses the majority of the endosomal sorting complex required for transport (ESCRT) machinery encoding genes, which participate in phagosome formation. Some of them are up- or down-regulated during phagocytosis [135,136]. The ESCRT-III (*E. histolytica* vacuolar protein sorting (EhVps)2, EhVps20, EhVps24 and EhVps32), and ESCRT-accessory proteins (EhVps4 and EhADH) have been isolated, characterized and some of them assembled in a giant unilamellar vesicular in vitro system, evidencing their participation in the multivesicular bodies and intraluminal vesicles formation [69,115,118,136].

Lipids coordinate signalling, targeting, and trafficking events during biogenesis and maturation of phagosomes. Phosphoinositides (PtdIns[3]P and PtdIns[4]P), EhFP4 (a FYVE domain-containing protein) and phosphoinositide 3-kinase (PI3K) signalling pathway components, harmonically regulate the phagocytosis beginning and phagosome maturation [116,117]. The importance of cholesterol and lysobisphosphatidic acid in phagocytosis has been also demonstrated during vesicles and phagolysosomes formation [59,137,138].

Unlike phagocytosis, trogocytosis imply pieces, but not entire cell engulfment, resulting in intracellular calcium elevation and eventual host cell death. This event requires close cell-cell contact and physiological temperature, and involves fast uptake rates [139]. Molecularly, trogocytosis provokes actin rearrangements, with the participation of the EhGal/GalNAc lectin, the *E. histolytica* C2 domain protein kinase (EhC2PK) kinase and PI3K signalling [140]. The AGC kinase EhAGCK1, which binds to PtdIns[3]P, has been involved in trogocytosis but not in phagocytosis of dead cells [141].

### 2.6. Host Immune Response

After the contact by trophozoites, the epithelium produces pro-inflammatory cytokines as interleukin (IL)-1β, IL-8 and tumour necrosis factor (TNF)-α to recruit neutrophils and macrophages to the infection site. The main amoebicidal activity of neutrophils is the release of reactive oxygen species (ROS) [142,143]. In macrophages, the EhGal/GalNAc lectin up-regulates the mRNA expression of different cytokines, modulates the TLR-2 receptor and NF-κB and MAPK pathways [144]. Macrophages activated with interferon (IFN)-γ or TNF-α kill trophozoites by producing nitric oxide (NO) [145]. Likewise, higher levels of IFN-γ are related to a lower incidence of *E. histolytica* infection [143,146]. Secreted EhPGE_2_ leads to the *il-8* mRNA expression in human colonic cells via EP_4_ receptor [147]. EhCPs also increase the expression of IL-8 via a protease activated receptor (PAR) 2-independent mechanism [148].

Recently, it has been described that the NACHT, LRR and PYD domains-containing protein 3 (NLRP3) inflammasome pathway are also activated after target cells make contact with live trophozoites [149]. The contact between *E. histolytica* and macrophages, triggers the recruitment of NLRP3 and α5β1 integrin activated by EhCP5 into the IJs [150]. Activation of inflammasome by *E. histolytica* leads to rapid and strong secretion of IL-1β, IL-18, IL-1α, FGF-2 and IP-10, but does not trigger the caspase-1 dependent cell death pathway [149].

The host immune response (innate and adaptive) against trophozoites invasion is strong, however *E. histolytica* survives by developing immune evasion strategies. Besides mucin-2, EhCPs induce the production and cutting of intestinal antimicrobial peptides as cathelicidins, which are important components of the innate immune defence [151]. In addition, Fe-superoxide dismutase (SOD) and a 29-kDa surface peroxiredoxin belonging to *E. histolytica* thiol-dependent redox metabolism system, and with potent antioxidant and redox regulatory roles, protects the parasite from the neutrophil reactive oxygen effects [67,152,153]. Trophozoites also induce neutrophil apoptosis through the activation of extracellular signal–regulated kinase (ERK) 1/2, by the generation of NADPH oxidase-derived ROS [154]. When *E. histolytica* is exposed to macrophages, the parasite cyclooxygenase-like enzyme synthetizes EhPGE_2_ [147], which inhibits the respiratory burst (ROS: H_2_O_2_, O^2−^, OH^−^) and NO production by macrophages [155,156]. Moreover, *E. histolytica* resists complement activation by the similarity and antigenic cross reactivity of the EhGal/GalNAc lectin with the host cluster of differentiation (CD)59 protein, inhibiting the complement-mediated lysis [157]. EhCPs also participate in the degradation of complement factors (C3 and C5), disruption of host adaptive response (IgA and IgG), and cleavage of ECM components (fibronectin, collagen and laminin) for a successful invasion, as mentioned in the cytolysis section [56,158,159,160].

### 2.7. Contributions of the Gut Microbiome

*Entamoeba* colonization is significantly affected by the microbiome composition and diversity. Indeed, the colonization can be predicted based on the composition of an individual’s gut microbiota with 79% accuracy [161]. Parasites and microbiome interact in different ways, altering the parasite virulence, inducing colonization resistance, provoking dysbiosis and modulating the host immunity [162]. Pioneer works demonstrated that the association of trophozoites with various types of gram-negative bacteria, noticeably increases the parasite virulence, as evidenced by their ability to destroy epithelial monolayers [163]. In addition, studies in gnotobiotic animals indicate that commensal bacteria are necessary for virulent *E. histolytica* infection [164]. Patients of northern India positive for *E. histolytica* presented dysbiosis, characterized by a reduction of *Bacteroides* spp., *Bifidobacterium* spp., *Ruminococcus* spp., *Lactobacillus* spp., *Clostridium leptum*, *Clostridium coccoides*, *Eubacterium* spp., *Campylobacter* spp. and *Methanobrevibacter smithii* in stool samples [165]. Furthermore, introduction of commensal bacteria alters the mucosal immune system and reduces the susceptibility of mice to *E. histolytica* colitis. Thus, alteration of the gut microbiota provides protection against infection by this parasite [162]. Conversely, an association between *Prevotella copri* and *Entamoeba* has been observed in infected children [166]. This bacterium associates with gut inflammation and the generation of an excessive immunity in patients and animal models [167], suggesting that parasite infection is influenced by the inflammatory state of the gut, which is potentially associated with changes in the gut microbiome [168].

In general, further microbiome studies may provide insights into why some patients get an invasive disease, which factors contribute to parasite-associated malnutrition and growth inhibition, and how *E. histolytica* may change gut flora [162,168].

## 3. *Naegleria fowleri*

Contaminated water, splashed or forced into the human nasal cavity, may produce *N. fowleri* infection, which initiates with trophozoites attachment to the nasal mucosa, followed by parasites locomotion along the olfactory nerve and the cribriform plate, and a chemotactic response to nerve cell components, to finally reach the CNS olfactory bulbs. There, *N. fowleri*, induces severe inflammation, as well as tissue necrosis and neurons destruction [171,172,173].

Pathogenic mechanisms of *N. fowleri* include adherence of trophozoites to nasal mucosa and mucin degradation. Afterwards, food-cups enable trophozoites to ingest human tissue, whereas secretion of naegleriapores, acid hydrolases, proteases, neuraminidases and phospholipases contribute to demyelination and further lysis of erythrocytes and surrounding nerve cells [174]. Trophozoites also display evasion mechanisms to the host immune response, such as the removal of the membrane attack complex and resistance to cytokines. *N. fowleri* pathogenicity and the overall intense host immune response, results in a significant nerve and CNS damage, and ultimately, to cell death [175] (Figure 2 and Table 2).

### 3.1. Penetration of Mucous Layer

During the initial steps of *N. fowleri* infection, the host response involves an immediate mucus secretion for trapping trophozoites [171]. However, *N. fowleri* has a variety of virulence factors to avoid the host response, including enzymes with mucinolytic activity. This activity has been reported for *Naegleria* spp. crude extracts and live trophozoites, and mucin effects on trophozoites is overcome in a time-dependent manner. Moreover, a 37 kDa NfCP of *N. fowleri* has an important role in mucin degradation and evasion of the host innate response [176].

### 3.2. Adherence of Trophozoites to the Host Epithelium

Adherence and direct contact of *N. fowleri* with target cells are crucial for parasite cytopathogenicity [177,178]. Carbohydrates containing mannose, glucose and fucose residues mainly participate in *N. fowleri* adherence and cytotoxicity to MDCK monolayers. Binding assays using different lectins, have shown the presence, composition and abundance of surface glycoconjugates containing α-d-glucosyl, α-d-mannosyl and α-l-fucosyl, *N*-acetyl-α-d-galactosaminyl and α-d-galactose residues in *Naegleria* species, and also differences between non-pathogenic *N. gruberi* and *N. lovaniensis*, and pathogenic *N. fowleri* trophozoites [174,179]. Although some glycoconjugates including glycolipids and glycoproteins are playing a role in *N. fowleri* attachment to nasal epithelium, this parasite may also use other molecules to follow host infection as lectins, adhesins and toxins [174]. However, there are few reports about their identity and biochemical characterization.

An *N. fowleri* integrin-like protein binds to immobilized fibronectin and has also a role in cytotoxicity [180]. Besides, *N. fowleri* interacts with human type I collagen [181], and a comparative study between pathogenic *N. fowleri* and non-pathogenic *N. lovaniensis* strains reveals greater adherence of *N. fowleri* to fibronectin, laminin-1, and collagen I, and the presence of two integrin-like proteins in focal adhesion-like structures [182]. 

### 3.3. Interaction with Epithelial Intercellular Junctions

The mechanism that enables *N. fowleri* penetration of the olfactory epithelium, and further invasion of CNS, is not completely understood. Previous observations reported neuroepithelium traversing by *N. fowleri* trophozoites without causing any apparent damage, thus suggesting the use of the paracellular route, presumably by TJs disruption [183]. Further evidence demonstrated TEER dropping with an increased epithelial permeability, confirming the parasite invasion through the paracellular route. Accordingly, morphological changes on the epithelium at early stages of *N. fowleri* interaction, leading to the disruption of ZO-1 and claudin-1, but not occludin, as well as actin apical ring alterations are observed [184]. Moreover, protozoans infecting the CNS migrate by the paracellular route and promote the production of cytokines, which disrupt the blood brain barrier by altering TJs. By using an in vitro blood brain barrier model, it has provided more accurate information about *N. fowleri* dissemination routes to the CNS. *N. fowleri* could damage the blood brain barrier by destabilizing TJs and activating endothelial cells, resulting in delocalization and degradation of claudin-5, occludin and ZO-1 in a time-dependent manner. Trophozoites also induce the expression of the pro-adhesion molecules vascular cell adhesion protein 1 (VCAM-1) and ICAM-1, provoking the recruitment of inflammatory cells and the production of IL-8, IL-1β, TNF-α, IL-6 and NO [185]. Trophozoites and their conditioned medium produce an important TEER decrease and cytopathic effects in rat brain microvascular endothelial cells (RBMEC), which results from NfCPs activity. *N. fowleri* can also modify the actin cytoskeleton and induce stress fibbers formation in RBMEC. Both TJs proteins degradation and actin alterations could promote inflammatory cells free passage from the peripheral blood circulation to the blood brain barrier [185]. Therefore, *N. fowleri* damage not only the olfactory epithelium but also endothelial cells (blood brain barrier) by altering IJs, although alternative pathways, such as the transcellular route, are not excluded. 

### 3.4. Cytolysis

Tissue invasion is achieved by the production of cytopathic enzymes that degrade and lyse mammalian cells and ECM [186,187]. In addition, it has evaluated the cytotoxic activity of *N. fowleri* cell-free extracts on rat cell cultures [188]. Factors so far identified include, among others, phospholipases, neuraminidase, elastase, naegleriapores, NfCPs and a cytopathic protein that triggers the apoptosis pathway in tissue culture cells [30,189,190,191].

The addition of the soluble proteins fraction, containing phospholipase A and phospholipase C activities, on nerve cells, results in immediate alterations of the cell surface, including cellular blebbing and holes in the plasma membrane [188]. Phospholipases, lysophospholipase and sphingomyelinase are lipolytic enzymes secreted in high levels in pathogenic *Naegleria,* hardly detectable in the culture media of virulent-attenuated and non-pathogenic species of *Naegleria* [191]. Similarly, neuraminidase activity is only detected in pathogenic *N. fowleri*. The activity of this enzyme is maximal at pH 4.5 and 5.0 and ion-independent and may contribute to the reported glycolipid alterations associated with demyelinating diseases [189]. Unlikely, the elastase is present in *N. fowleri* and in other species that lost pathogenicity as a result of long-term axenic maintenance [187].

The enormous cytolytic activity of intact amoebae, as well as of crude extracts, against a variety of target cells, is in part attributed to naegleriapores, that in contrast to amoebapores, organize as several isoforms in a pre-promultipeptide structure, possibly to synthesize a wide variety of molecules for killing phagocytosed bacteria, or facing effector cells from the host immune system [190].

*N. fowleri* CPs participate in PAM progression, as demonstrated by using 34 specific inhibitors of CPs activity in cell lysates and analysing their effect on parasite viability. It also results in a significant decrease of NfCPs activity in vitro, and dead or non-dividing trophozoites [192]. The *N. fowleri* cathepsin B (NfCPB) and cathepsin B-like (NfCPB-L) proteins are initially transcribed as longer precursor molecules that undergo amino acids cleavage, to generate the 25 kDa and 24 kDa active forms, respectively. These proteins are localized on cell membranes, pseudopodia and food-cup structures, and may be easily secreted. Both enzymes display proteolytic activity on immunoglobulins, collagen, fibronectin, haemoglobin and albumin. Particularly, the *cathepsin B* gene is expressed in the trophozoite, but not in the cyst stage [193]. In addition to the aforementioned 37 kDa NfCP with mucinolytic activity, *N. fowleri* trophozoites also secrete a 30 kDa NfCP that produces a cytopathic effect on baby hamster kidney fibroblasts (BHK) cells [186].

### 3.5. Phagocytosis and Trogocytosis

*N. fowleri* phagocytoses and trogocytoses a variety of cultured mammalian cells, both processes related to the extension of food-cups or amoebastomes [39,177,178]. Although there is not a definitive correlation between the presence of food-cups on amoebae and in vivo pathogenicity, these structures are rarely observed in non-pathogenic *N. gruberi* or *N. lovaniensis* [194,195,196].

The *N. fowleri* antigen-related protein 1 (Nfa1) protein is abundant in *N. fowleri* pseudopodia contacting target cells, food-cups formed as phagocytic structures during ingestion of target cell debris or lysed material, and around food vacuoles, thus, associating this protein to amoebic movement and food ingestion [197]. Alternatively, cytolytic molecules may be released at the attachment site between food-cups and target cells [188].

Both, phagocytosis and trogocytosis, depend on the dynamics of cytoskeleton rearrangements. By immunofluorescence assays, myosin and tubulin have been detected in a dispersed pattern within trophozoites, whereas actin is cytosolic and also present in pseudopodia and food-cup structures [198]. The *N. fowleri* actin (Nfactin) shows 82% identity to the non-pathogenic *N. gruberi* actin corresponding sequence, and it does not have any identity to human actin. Nfactin localizes in cytoplasm, pseudopodia, and especially, food-cup structures. Moreover, *N. fowleri* trophozoites in co-culture with CHO cells, strongly express Nfactin on food-cup structures concerning trogocytosis. Conversely, actin polymerization inhibitors, such as cytocalasin D, or *Nfactin* gene silencing, reduce food-cup formation and in vitro cytotoxicity, suggesting a decreased ability of trophozoites to attach to cells, phagocyte them, and thus, to produce damage [199].

### 3.6. Host Immune Response

Epithelium invasion by trophozoites, induces an increasing inflammatory process with eosinophils and neutrophils participation [171,183]. Neutrophils surround parasites before killing them, by contact-dependent and independent mechanisms. TNF-α stimulates neutrophils adherence to *N. fowleri* and increases their activity by enhancing oxygen radical’s production. Neutrophils are also capable of destroying *N. fowleri* by pinching off and engulfing portions of trophozoites [200,201,202]. Selective neutrophils depletion leads to increased mortality in immunized mice, after challenges with *N. fowleri,* whereas no treatment produces 95% of mice survival [203]. The host inflammatory response and polymorphonuclear cell lysis contribute mainly to CNS damage [183]. Microglial cells exposed to *N. fowleri* lysates, release TNF-α, IL-6, and IL-1β, whereas astrocytes lead to the expression of IL-8, IL-α, and IL-6 in an ERK, c-jun N-terminal kinase (JNK) and p38 mitogen-activated protein kinase (MAPK)-dependent pathways [204].

Activated macrophages kill *N. fowleri* trophozoites by producing NO, non-oxidative mediators, such as TNF-α and IL-1, and possibly other factors. Likewise, natural killer cells exhibit an amoebicidal activity that increases during the course of the infection, hence, enhancing parasites killing [200,205,206,207].

Meanwhile, *N. fowleri* evasion to the host immune response includes mucinolytic activity [176], and resistance to complement-mediated lysis [208]. Chelation of extracellular calcium enhances complement-mediated lysis of *N. fowleri*, suggesting a role for Ca^2+^ in complement resistance [209]. In addition, *N. fowleri* resists complement damage due to the expression of complement regulatory proteins, such as a the CD59-like surface protein [210,211], and shedding of the membrane attack complex on vesicles [212]. Indeed, the enzymatic removal of trophozoites surface components increases their susceptibility to complement-mediated lysis [212], as well as the blocking of protein kinases activity, which have been associated with complement resistance [213]. The ability of *N. fowleri* to internalize surface-bound antibodies also contributes to the parasite evasion of the host immune system, for instance, by capping and internalizing sIgA antibodies [181,214].

## 4. *Acanthamoeba* spp.

Once *Acanthamoeba* enters the eye to produce AK, it adheres to the corneal epithelium, triggering parasite-mediated cytolysis and phagocytosis, and eventually induces programmed cell death [42,215]. Pathogenesis of GAE is complex since hematogenous spread is a pre-requisite for blood brain barrier invasion by amoebae, to finally enter the CNS [30,41,216].

To produce infection, *Acanthamoeba* requires (i) the expression of adhesins, the production of toxins and the ability to resist the immune and environmental factors, as well as chemotherapeutic agents [40,44]; (ii) the production of food-cups or amoebostomes which help in nibbling away bits and pieces of tissue surface, similar to trogocytosis presented by *E. histolytica* [217,218]; and (iii) the secretion of enzymes such as phospholipases, lysosomal hydrolases, aminopeptidases, SPs, and metalloproteinases, as well as plasminogen activators [38,40,44,219,220,221] (Figure 3 and Table 3).

### 4.1. Adherence of Trophozoites to the Host Epithelium

Pathogenic *Acanthamoeba* interacts with host cells through the glycocalyx, the main carbohydrate-containing component of the cell membrane [41,44]. Thus, several adhesins including a >207 kDa adhesin [222], a mannose-binding protein (MBP) [223] and a laminin-binding protein (AhLBP) [224], contribute to trophozoites attachment by interacting with glycoproteins and glycolipids present on the corneal surface [44].

The >207 kDa adhesin, identified by adherence-associated antibodies, is a surface membrane glycoprotein that does not bind to mannose, and participates in the *A. castellanii* adherence to hamster corneal epithelial cells [222].

MBP (136 kDa) harbours in its N-terminal extracellular domain five *N*- and three *O*-glycosylation sites, a transmembrane domain, and a C-terminal intracellular domain. It is expressed at the trophozoites membrane and facilitates parasite adherence to mannosylated proteins at the epithelium [223,225]. Moreover, exogenous free α-mannose inhibits by 80% the binding of *Acanthamoeba* to the epithelium, whereas lactose, galactose and fucose do not. This inhibition correlates with an increase of the protease’s secretion, suggesting that MBP stimulates the expression and secretion of proteases. MBP is also crucial for trophozoites adherence to ECM components, such as collagen, fibronectin and laminin [226]. In agreement, *A. castellanii* produces a cytolytic mannose-induced protein of 133 kDa (MIP133) after exposure to the upregulated mannose-specific lectins in the ulcerated corneal epithelium, that leads to the activation and upregulation of MMPs in corneal cells [227]. In addition, MIP133 degrades both human types I and IV collagen [225].

Besides, *A. healyi* adhere via AhLBP (55 kDa) to the Bowman’s layer and ECM components (laminin, collagen type I), which underlie the basal lamina [224,228]. Similarly, *A. polyphaga* binds to laminin, collagen IV and fibronectin in a mannose- and calcium-dependent manner [229,230]. Thus, binding to laminin and collagen has been recognized as an attribute of pathogenic *Acanthamoeba* species [231].

Furthermore, by using cytochalasin B and latrunculin B, it has been revealed the participation of cytoskeletal filaments in *A. castellanii* adherence to epithelial and neuronal cells [44].

### 4.2. Interaction with Epithelial IJs

After adhering to the epithelium, *Acanthamoeba* trophozoites place close to IJs to destabilize their barrier function [232,233,234]. *A. culbertsoni* trophozoites isolated from a severe case of AK are introduced through the intercellular space into the MDCK monolayers, suggesting that IJs are affected. In fact, this parasite drops TEER by 40%, highlighting a disruption of TJs [235]. Interestingly, integrity of TJs is restored after amoebae cross the monolayer, suggesting a reversible effect on the junctional disruption. Moreover, *A. castellanii* migrates between corneal cell layers, however, no evidence of cell injury is observed. These results suggest a mechanical action of parasites promoting cellular separation, but not cell lysis at the beginning of interaction [218]. On the other hand, *A. royreba* also decreases TEER values close to zero after interaction with human brain microvascular endothelial cells (HBMEC), inducing a Rho-dependent reduction of occludin and ZO-1 [233]. *Acanthamoeba* spp. may be modifying the epithelium from the cornea and the blood brain barrier in dissimilar ways, probably due to differences in TJs composition.

Proteases also seem to be involved in TJs injury, since *A. castellanii* trophozoites pre-incubated with phenylmethylsulfonyl fluoride (PMSF) and E-64 (specific inhibitors for SPs and CPs, respectively) avoid the mislocalization of ZO-1 and occludin. Moreover, *Acanthamoeba* secretes large amounts of SPs [236] that degrade ECM components (collagen I, III and IV, elastin and fibronectin), fibrinogen, IgA, IgG, albumin, plasminogen and haemoglobin [220,237]. Indeed, extracellular proteases have been related to HBMEC monolayer disruptions, facilitating the trophozoites migration from systemic circulation to the CNS tissues. Besides, the presence of *Acanthamoeba* at the lamina propria in the blood brain barrier [169], suggests that this parasite may also damage AJs.

### 4.3. Cytolysis

*Acanthamoeba* spp. can destroy cells by either direct or indirect cytolysis of target cells. The exact mechanisms involved in trophozoite-mediated cytolysis are not known, but destruction of target cells occur by apoptosis [215,238]. Few parasite molecules have been related with cytolysis, such as SPs [97,239,240], CPs [97,240], metalloproteases [236], and cytotoxic proteases induced by mannose mediated adhesion [241].

The proteinases work in concert to produce a potent cytopathic effect, involving host cell killing, degradation of the epithelial basement membrane and underlying stromal matrix, and penetration into the deeper layers of the cornea [242]. Overall, several studies have implicated proteases with *Acanthamoeba* pathogenicity, being also salient to survival, multiplication, differentiation and cellular transformation [97,220,236,243]. Crude extracts of *A. polyphaga* display at least seven proteases ranging from 34 to 144 kDa, whereas those of *A. castellanii* exhibit at least nine different proteases from 30 to 188 kDa. By using protease inhibitors, it was demonstrated that the SPs are the most abundant, followed by the CPs [244]. Subsequent to the MBP-mediated adherence to host cells, trophozoites produce a contact-dependent metalloproteinase and several contact-independent SPs [226,242].

*Acanthamoeba* spp. secrete SPs of various molecular weights (12, 40, 42, 55, 70, 85, 97, 107, 130, 133 and 230 kDa), all sensitive to the PMSF inhibitor [220]. The *Acanthamoeba* plasminogen activator (aPA) is a SP that facilitates the invasion of *A. castellanii* trophozoites to the host and contributes to the pathogenesis of AK. In human corneal epithelial cells, aPA induces expression and production of IL-8 via the PARs pathway [245]. *Acanthamoeba* CPs are involved in the proteolytic degradation of cellular components, including iron-binding proteins, and also play important roles for encystation [243,244,246,247]. Nevertheless, so far, only three cathepsin L-like CPs, AcCP2, AhCP and AcCP, have been identified in *A. culbertsoni*, *A. healyi* and *A. castellanii*, respectively [246,248,249]. The metalloprotease activity against collagen I and III, elastin and plasminogen, results in the degradation of the basement membranes. Additionally, other enzymes such as collagenases and elastases are constitutively produced by pathogenic *Acanthamoebae* and contribute to cytolysis [220].

In the case of MBP participation in amoebic cytotoxicity, evidences remain contradictory. The inhibition of *A. castellanii* adherence to corneal epithelial cells by using mannose, results in the loss of trophozoites to produce cytotoxicity [226], while in another work, it is exacerbated [241].

MIP133 is secreted to facilitate the degradation or corneal layers, leading to parasite’s infiltration around the corneal nerves, causing radial neuritis and pain [225]. MIP133 induces the degradation of keratocytes, iris ciliary body cells, retinal pigment epithelial cells, corneal epithelial cells and corneal endothelial cells, and leads to apoptosis of macrophage-like cells [225,241]. 

In a similar way to amoebapores and naegleriapores, *Acanthamoeba* spp. express pore-forming proteins as part of their cytolytic armamentarium, switching from a soluble form to a membrane-inserted state. However, acanthaporins display structural differences and do not resemble the same protein folding of amoebapores and naegleriapores [250]. 

### 4.4. Phagocytosis

By phagocytosis, *Acanthamoeba* ingests host epithelial cells and food particles. Bound particles are surrounded by pseudopods, brought into the cytoplasm and released as phagosomes [215]. Parasite engulfment of corneal epithelial cells is mediated by food-cups or amoebastomes, structures that have been also observed and implicated in *E. histolytica* and *N. fowleri* pathogenesis [251].

During phagocytosis, actin-mediated cytoskeletal rearrangements play an important role for *Acanthamoeba* pathogenesis, as demonstrated by using cytocalasin D, an inhibitor for actin polymerization, and phosphatase and kinase inhibitors, which points to the tyrosine-kinase-induced actin polymerization pathway [252]. Additionally, a Rho kinase inhibitor, Y27632, which blocks stress fibber formation, partially prevents phagocytosis. Similarly, LY294002, a specific inhibitor of PI3K, inhibits *Acanthamoeba* phagocytosis, and inhibition of Src kinase using PP2, also hampers the phagocytic ability of *A. castellanii* [215,251].

Evidence, including host cell DNA laddering, chromatin condensation, membrane blebbing and formation of apoptotic bodies, has suggested that *Acanthamoeba* produces apoptosis in neuroblastoma cells, represent an alternative mechanism for host cell death [253]. However, whether apoptosis and phagocytosis have independent roles in *Acanthamoeba* pathogenesis or apoptosis is a primary process stimulated by initial binding of parasites to the host cells that eventually leads to secondary events, such as phagocytosis, remains to be determined [216,238].

### 4.5. Host Immune Response

Innate immune responses are considered the most important host defences against *Acanthamoeba*, according to histopathology of AK, which is characterized by the presence of macrophages, neutrophils, and few lymphocytes [254].

*Acanthamoeba* trophozoites co-cultured with microglial cells produce a variety of interleukins including IL-1α and β, and TNF. Moreover, Toll-like receptor 4 (TLR4) is upregulated in human and Chinese hamster corneal epithelial cells upon *Acanthamoeba* stimulation, leading to a significant increase of proinflammatory chemokines production, including chemokine (C-X-C motif) ligand 2 (CXCL2) [255]. Interleukins, together with macrophages infiltration might play a role in the clearance of *Acanthamoeba* by the host. Macrophage-mediated killing is probably contact-dependent. Additionally, neutrophils might release lysosomal enzymes and ROS (HOCl and H_2_O_2_) after activation by the complement, thereby killing the amoebae. However, in immunosuppressed individuals, due to the lack of T-lymphocytes and macrophages, and the impairment of cell-mediated immunity, *Acanthamoeba* proliferates and damages the CNS and other tissues [254,256]. Anti-acanthamoebic IgA antibodies inhibit parasite binding to host cells, block the secretion of cytotoxic substances and play a crucial role in the ultimate macrophage-mediated complement lysis, that provides protection against AK. However, pathogenic *Acanthamoeba* can resist macrophage-mediated complement lysis [257].

This parasite binds the C1q component of the complement pathway, which under normal conditions, provides the basis for opsonin and phagocytosis processes [258]. Additionally, trophozoites can degrade IgG and IgA antibodies, indicating their potential to interfere with the immune system, and revealing the need for developing effective therapeutic interventions [259,260].

On the other hand, superoxide dismutases (SODs) are important antioxidant defences exposed to oxygen [261]. A 50 kDa iron and a 38 kDa copper-zinc SODs have been reported as potential virulence factors for *Acanthamoeba*, acting as anti-oxidants and anti-inflammatory agents [262,263]. *A. castellanii* iron SOD may play an essential role in trophozoites survival by protecting them from endogenous oxidative stress, but also by detoxifying oxidative killing of parasites by the host immune cells [263].

## 5. Concluding Remarks

Pathogens causative of human disease mainly comprise viruses, bacteria and protozoa. After infection with any of them, a battle between the host immune system and the virulence mechanisms of the parasite is established. The recovery of health entails the achievement of host homeostasis, depending on the nature and severity of the infection, and the existence of prophylactic or therapeutic interventions. To invade and colonize the host, pathogens have deployed diverse tactics, such as the successful exploitation of host epithelial cells and molecules to reach target tissues, and the evasion of the host immune response.

Epithelia are the tissues most exposed to the environment and they prevent the invasion by pathogenic microorganisms and their products, acting as a mechanical barrier. Additionally, physical cleansing effects of mucous secretions and shedding of colonized cells normally contribute to protect against microorganisms. Conversely, most pathogens have developed mechanisms to disrupt epithelial layers and open their way to other tissues and organs.

This review outlines the importance of epithelia and IJs in invasion by virus, bacteria and protozoa, and focuses on events associated to the interaction of three pathogenic amoebae *E. histolytica*, *N. fowleri* and *Acanthamoeba* spp. with the host intestinal, olfactory or corneal epithelium, respectively. These protozoa display a high motility to achieve their progression across tissues, surmounting the barrier function to invade other organs by using several molecules. Some of them are present at the parasites surface and others are secreted by trophozoites to exert their functions efficiently.

In the case of the pathogenic amoebae, the key events leading to epithelial damage include the penetration of the mucous layer, adherence to target cells, interaction with IJs, as well as cell lysis and engulfment.

To penetrate epithelial cells, amoebae utilize the transcellular and paracellular pathways. They have evolved specific strategies for exploiting molecules present in the epithelium, mainly by contacting, dislocating and degrading IJs proteins, which protect and maintain the epithelial integrity. Moreover, some parasite proteins exhibit structural similarities to host proteins, thus mimicking them, competing and displacing them during invasion. These alterations in IJs proteins alternatively produce cytoskeleton rearrangements and trigger signalling cascades for the up- and down-regulation of effector proteins in the host, as a response to parasite virulence factors. Immediately, injured epithelial cells initiate the inflammatory response by emitting chemotactic signals that attract blood-borne host defence cells. However, this response also exacerbates cellular and tissue damage. In all these events, *E. histolytica*, *N. fowleri* and *Acanthamoeba* spp. express adhesins, lectins, pore-forming proteins, cysteine and serine proteases, hydrolases, glycosidases, actin-binding proteins, signaling factors and several other molecules.

Analysis of the virulence mechanisms of these amoebae and other microorganisms reinforce the fact that pathogens still continue to evolve and emerge. Arguably, the most successful strategies for parasite survival will be those that exploit their hosts without killing them. These activities are modulated by the interaction of panoply of virulence factors with their cognate host cell receptors, and signals are bidirectionally sent from the microbial pathogen to the host and from the host to the pathogen at multiple stages of the invasion process. Recent advances place us on the verge of understanding this complex process in detail, at molecular level. With the accelerated integration of tools from cell biology, biochemistry, biophysics, and genomics, in the next few years, the experimental approaches should bring unprecedented insights into the interactions of pathogenic amoebae with their host, thus, leading further to the design of novel therapeutic drugs against these parasites.

## Figures and Tables

**Table 1:** Parasite molecules from *E. histolytica* involved in host invasion.

Parasite Molecules	Host Receptor/Effector	References
EhGal/GalNAc lectin	mucin, glycoproteins, ECM components, TLR-2 receptor, NF-κΒ, MAPK, cytokines, CD59	[48,51,76]
220 kDa lectin	hyaluronic acid, chitin, chitotriose, *N*-acetyl-galactosamine, galactose	[64,65]
Glycosidases (sialidase, *N*-acetyl-galactosamidase, *N*-acetyl glucosaminidase)	mucin	[50]
EhCPADH	occludin, claudin-1, ZO-1, ZO-2, E-cadherin, β-catenin, desmoplakin I/II	[72,82]
EhADH	97 kDa putative receptor	[100]
EhCP112	mucin, gelatine, collagen, fibronectin, haemoglobin, occludin, claudin-1, claudin-2, ZO-1, ZO-2, E-cadherin, β-catenin, desmoglein-2, desmoplakin I/II	[54,72]
EhCP1	villin, collagen, C3, IgG, pro-IL-18, pro-IL-1β, cathelicidins	[85,89,97,151,169]
EhCP2	villin, collagen, IgA, IgG, chemokines, C3, pro-IL-18	[90]
EhCP4	villin	[85,89]
EhCP5	mucin, collagen, IgA, pro-IL-18, haemoglobin, fibrinogen, BSA, α5β1 and αvβ3 integrins, NLRP3, cytokines, pro-MMP3	[55,62,103]
EhSPs	villin, ZO-1, ZO-2	[85]
EhROM1	unknown host transmembrane or peripheral proteins	[78]
EhPGE_2_	EP_4_ receptor, claudin-4, IL-8, ROS, NO	[86]
Occludin-like protein	occludin	[87]
KERP1	unknown	[66,79]
PFO	unknown	[80]
Amoebapores A, B and C	host cell membranes	[96]
EhTMKs (EhTMK39, EhTMKB1-9, EhPATMK)	unknown	[120]
EhMSP-1	unknown	[122]
ABPs (ARPC1, NCABP166, myosin IB)	unknown	[170]
EhCaBPs	unknown	[113]
Small GTPases (EhRabA, EhRabB, EhRab5, EhRab7A, EhRab7B, EhRab8A, EhRab11B, p21RacA)	unknown	[114,125]
ESCRT-III (EhVps2, EhVps20, EhVps24, EhVps32)	unknown	[115]
EhVps4	unknown	[136]
Phosphoinositides (PtdIns[3]P, PtdIns[4])	unknown	[117]
Cholesterol	unknown	[59]
Lysobisphosphatidic acid	unknown	[137]
Fe-SOD 29 kDa peroxiredoxin	ROS	[67,152,153]

EhGal/GalNAc: *E. histolytica* d-galactose and *N*-acetyl-d-galactosamine; EhCP112: *E. histolytica* 112 kDa cysteine protease; EhADH: *E. histolytica* adhesin; EhCPADH: *E. histolytica* complex formed by EhCP112 and EhADH; EhCP: *E. histolytica* cysteine protease; EhSPs: *E. histolytica* serine proteases; EhROM1: EhCP: *E. histolytica* rhomboid protein 1; EhPGE_2_: *E. histolytica* prostaglandin E_2_; KERP1: lysine and glutamic acid enriched protein; PFO: pyruvate:ferredoxin oxidoreductase; EhTMKs: *E. histolytica* transmembrane kinase family proteins; EhPATMK: *E. histolytica* phagosome-associated TMK96 protein; EhMSP-1: *E. histolytica* metallosurface protease 1; ABPs: actin binding proteins; ARPC1: actin-related protein 2/3 complex subunit 1; NCABP166: 166 kDa nucleocytoplasmic actin-binding protein; EhCaBPs: *E. histolytica* calcium binding proteins; ESCRT-III: endosomal sorting complex required for transport; EhVps: *E. histolytica* vacuolar protein sorting; SOD: superoxide dismutase; ECM: extracellular matrix; TLR-2: Toll-like receptor 2; MAPK: p38 mitogen-activated protein kinase; CD59: host cluster of differentiation 59 protein; ZOs: *zonula occludens* proteins; C3: complement factor 3; Ig: immunoglobulins; IL: interleukin; BSA: bovine serum albumin; NLRP3: NACHT, LRR and PYD domains-containing protein 3; pro-MMP3: pro-matrix metalloproteinase 3; EP_4_: prostaglandin E_2_ receptor 4; ROS: reactive oxygen species; NO: nitric oxide.

**Table 2:** Parasite molecules from *N. fowleri* involved in host invasion.

Parasite Molecules	Host Receptor/Effector	References
Mucinolytic enzymes	mucin	[176]
Glycoproteins	unknown	[179]
Glycolipids	unknown	[179]
Lectins	unknown	[174,179]
Adhesins	unknown	[174]
Integrin-like protein	fibronectin	[180]
NfCPs	claudin-1, ZO-1	[184]
37 kDa NfCP	mucin	[176]
30 kDa NfCP	unknown	[186]
NfCPB, NfCPB-L	immunoglobulins, collagen, fibronectin, haemoglobin, albumin	[193]
Nfa1	unknown	[197]
Myosin, tubulin, Nfactin	unknown	[198]
Neuraminidase, elastase	elastin	[187,189]
Lipolytic enzymes (phospholipases, lysophospholipase, sphingomyelinase)	unknown	[191]
Naegleriapores	host cell membranes	[190]
CD59-like protein	complement	[210]

NfCPs: *N. fowleri* cysteine proteases; NfCPB: *N. fowleri* cathepsin B; NfCPB-L: *N. fowleri* cathepsin B-like.

**Table 3:** Parasite molecules from *Acanthamoeba* spp. involved in host invasion.

Parasite Molecules	Host Receptor/Effector	References
Adhesins (>207 kDa adhesin, MBP, AhLBP)	glycoproteins, glycolipids, collagen, fibronectin, laminin	[222,223,226,228]
MIP133	collagens I and IV; MMP-2 and -3	[225,227]
Actin	unknown	[44]
SPs	occludin, ZO-1, collagens, elastin, fibronectin, fibrinogen, IgA, IgG, albumin, plasminogen, haemoglobin	[220,237,239]
aPA	IL-8, PAR	[245]
CPs	occludin, ZO-1, iron-binding proteins	[243,247]
AcCP2, AhCP, AcCP	unknown	[246,248,249]
Acanthaporin	host cell membranes	[250]
Metalloproteases	collagens, elastin, plasminogen	[220]
Collagenases	collagens	[220]
Elastases	elastin	[220]
Iron and copper-zinc SOD	ROS	[262,263]

MBP: mannose-binding protein; AhLBP: laminin-binding protein; MIP133: cytolytic mannose-induced protein of 133 kDa; MMP: pro-matrix metalloproteinase; SPs: serine proteases; aPA: *Acanthamoeba* plasminogen activator; PAR: protease activated receptor; CPs: cysteine proteases.

## References

[1] Dye C. (2014). After 2015: Infectious diseases in a new era of health and development. Philos. Trans. R. Soc. Lond. B. Biol. Sci..

[2] NIH Emerging and Re-Emerging Infectious Diseases-Developed under a Contract from the National Institutes of Health in collaboration with the National Institute of Allergy and Infectious Diseases. Biological Sciences Curriculum Study (BSCS). https://science.education.nih.gov/supplements/nih1/Diseases/guide/pdfs/nih_diseases.pdf.

[3] Lowe J.S., Anderson P.G. (2015). Chapter 3–Epithelial Cells. Stevens & Lowe’s Human Histology.

[4] Capaldo C.T., Farkas A.E., Nusrat A. (2014). Epithelial adhesive junctions. F1000Prime Rep..

[5] Liang G.H., Weber C.R. (2014). Molecular aspects of tight junction barrier function. Curr. Opin. Pharmacol..

[6] Lingaraju A., Long T.M., Wang Y., Austin J.R., Turner J.R. (2015). Conceptual barriers to understanding physical barriers. Semin. Cell Dev. Biol..

[7] Harris T.J.C., Tepass U. (2010). Adherens junctions: From molecules to morphogenesis. Nat. Rev. Mol. Cell Biol..

[8] Garrod D., Chidgey M. (2008). Desmosome structure, composition and function. Biochim. Biophys. Acta.

[9] Hartsock A., Nelson W.J. (2008). Adherens and tight junctions: Structure, function and connections to the actin cytoskeleton. Biochim. Biophys. Acta.

[10] Getsios S., Amargo E.V., Dusek R.L., Ishii K., Sheu L., Godsel L.M., Green K.J. (2004). Coordinated expression of desmoglein 1 and desmocollin 1 regulates intercellular adhesion. Differentiation.

[11] Citi S. (2018). Intestinal barriers protect against disease. Science.

[12] Yoo S.-J., Ryu S., Kim S., Golebiowski J., Han H.S., Moon C. (2017). Encyclopedia of the Neurological Sciences.

[13] Gerhard D. (2013). Neuroscience. 5th Edition. Yale J. Biol. Med..

[14] DelMonte D.W., Kim T. (2011). Anatomy and physiology of the cornea. J. Cataract Refract. Surg..

[15] Sridhar M.S. (2018). Anatomy of cornea and ocular surface. Indian J. Ophthalmol..

[16] Roman C., Solh T., Broadhurst M. (2017). Infectious Diarrhea. Physician Assist. Clin..

[17] Di Genova B.M., Tonelli R.R. (2016). Infection strategies of intestinal parasite pathogens and host cell responses. Front. Microbiol..

[18] Kazmierczak B.I., Mostov K., Engel J.N. (2001). Interaction of bacterial pathogens with polarized epithelium. Annu. Rev. Microbiol..

[19] Guttman J.A., Finlay B.B. (2009). Tight junctions as targets of infectious agents. Biochim. Biophys. Acta.

[20] Nikitas G., Cossart P., Harris T. (2012). Adherens Junctions and Pathogen Entry. Subcell Biochem.

[21] Hussein H.A.M., Walker L.R., Abdel-Raouf U.M., Desouky S.A., Montasser A.K.M., Akula S.M. (2015). Beyond RGD: Virus interactions with integrins. Arch. Virol..

[22] Basnet S., Palmenberg A.C., Gern J.E. (2019). Rhinoviruses and their receptors. Chest.

[23] Lublin D.M. (2005). Review: Cromer and DAF: Role in health and disease. Immunohematology.

[24] Nava P., López S., Arias C.F., Islas S., González-Mariscal L. (2004). The rotavirus surface protein VP8 modulates the gate and fence function of tight junctions in epithelial cells. J. Cell Sci..

[25] Abadi A.T.B. (2017). Strategies used by helicobacter pylori to establish persistent infection. World J. Gastroenterol..

[26] Saadat I., Higashi H., Obuse C., Umeda M., Murata-Kamiya N., Saito Y., Lu H., Ohnishi N., Azuma T., Suzuki A. (2007). Helicobacter pylori CagA targets PAR1/MARK kinase to disrupt epithelial cell polarity. Nature.

[27] Hodges K., Gill R. (2010). Infectious diarrhea: Cellular and molecular mechanisms. Gut Microbes.

[28] Siddiqui R., Ali I.K.M., Cope J.R., Khan N.A. (2016). Biology and pathogenesis of Naegleria fowleri. Acta Trop..

[29] Kantor M., Abrantes A., Estevez A., Schiller A., Torrent J., Gascon J., Hernandez R., Ochner C. (2018). Entamoeba Histolytica: Updates in Clinical Manifestation, Pathogenesis, and Vaccine Development. Can. J. Gastroenterol. Hepatol..

[30] Visvesvara G.S. (2010). Amebic meningoencephalitides and keratitis: Challenges in diagnosis and treatment*. Curr. Opin. Infect. Dis..

[31] Lozano R., Naghavi M., Foreman K., Lim S., Shibuya K., Aboyans V., Abraham J., Adair T., Aggarwal R., Ahn S.Y. (2012). Global and regional mortality from 235 causes of death for 20 age groups in 1990 and 2010: A systematic analysis for the Global Burden of Disease Study 2010. Lancet.

[32] Kissoon-Singh V., Moreau F., Trusevych E., Chadee K. (2013). Entamoeba histolytica exacerbates epithelial tight junction permeability and proinflammatory responses in Muc2(-/-) mice. Am. J. Pathol..

[33] Chalmers R.M., Percival S.L., Yates M.V., Williams D.W., Chalmers R.M., Gray N.F. (2014). Chapter Eighteen—Entamoeba Histolytica.

[34] Stanley S.L. (2003). Amoebiasis. Lancet.

[35] CDC Parasites—Protozoa. https://www.cdc.gov/parasites/.

[36] Grace E., Asbill S., Virga K. (2015). *Naegleria fowleri*: Pathogenesis, diagnosis, and treatment options. Antimicrob. Agents Chemother..

[37] Baig A.M., Khan N.A. (2015). Tackling infection owing to brain-eating amoeba. Acta Trop..

[38] Schuster F.L., Visvesvara G.S. (2004). Free-living amoebae as opportunistic and non-opportunistic pathogens of humans and animals. Int. J. Parasitol..

[39] Ma P., Visvesvara G.S., Martinez A.J., Theodore F.H., Daggett P.M., Sawyer T.K. (1990). Naegleria and Acanthamoeba infections: Review. Clin. Infect. Dis..

[40] Marciano-Cabral F., Cabral G. (2003). *Acanthamoeba* spp. as agents of disease in humans. Clin. Microbiol. Rev..

[41] Chalmers R.M., Percival S.L., Yates M.V., Chalmers R.M., Gray N.F. (2014). Chapter Fourteen—Acanthamoeba. Microbiology of Waterborne Diseases.

[42] Carvalho F.R.S., Foronda A.S., Mannis M.J., Höfling-Lima A.L., Belfort R., De Freitas D. (2009). Twenty years of *Acanthamoeba* keratitis. Cornea.

[43] Visvesvara G.S., Garcia H.H., Tanowitz H.B., Del Brutto O. (2013). Infections with free-living amebae. Neuroparasitology and Tropical Neurology.

[44] Soto-Arredondo K.J., Flores-Villavicencio L.L., Serrano-Luna J.J., Shibayama M., Sabanero-López M. (2014). Biochemical and cellular mechanisms regulating *Acanthamoeba castellanii* adherence to host cells. Parasitology.

[45] Martinez-Palomo A., Gonzalez-Robles A., Chavez B., Orozco E., Fernandez-Castelo S., Cervantes A. (1985). Structural bases of the cytolytic mechanisms of *Entamoeba histolytica*. J. Protozool..

[46] Anaya-Velazquez F., Martinez-Palomo A., Tsutsumi V., Gonzalez-Robles A. (1985). Intestinal invasive amebiasis: an experimental model in rodents using axenic or monoxenic strains of *Entamoeba histolytica*. Am. J. Trop. Med. Hyg..

[47] Christy N.C.V., Petri W.A. (2011). Mechanisms of adherence, cytotoxicity and phagocytosis modulate the pathogenesis of *Entamoeba histolytica*. Future Microbiol..

[48] Chadee K., Petri W.A., Innes D.J., Ravdin J.I. (1987). Rat and human colonic mucins bind to and inhibit adherence lectin of *Entamoeba histolytica*. J. Clin. Invest..

[49] Bruchhaus I., Loftus B.J., Hall N., Tannich E. (2003). The intestinal protozoan parasite *Entamoeba histolytica* contains 20 cysteine protease genes, of which only a small subset is expressed during in vitro cultivation. Eukaryot Cell.

[50] Thibeaux R., Weber C., Hon C.C., Dillies M.A., Avé P., Coppée J.Y., Labruyère E., Guillén N. (2013). Identification of the Virulence Landscape Essential for *Entamoeba histolytica* Invasion of the Human Colon. PLoS Pathog..

[51] Petri W.A., Haque R., Mann B.J. (2002). The bittersweet interface of parasite and host: lectin-carbohydrate interactions during human invasion by the parasite *Entamoeba histolytica*. Annu. Rev. Microbiol..

[52] Yadav R., Verma K., Chandra M., Mukherjee M., Datta S. (2016). Biophysical studies on calcium and carbohydrate binding to carbohydrate recognition domain of Gal/GalNAc lectin from *Entamoeba histolytica*: Insights into host cell adhesion. J. Biochem..

[53] Bruchhaus I., Jacobs T., Leippe M., Tannich E. (1996). *Entamoeba histolytica* and *Entamoeba dispar*: Differences in numbers and expression of cysteine proteinase genes. Mol. Microbiol..

[54] Cuellar P., Hernández-Nava E., García-Rivera G., Chávez-Munguía B., Schnoor M., Betanzos A., Orozco E. (2017). *Entamoeba histolytica* EhCP112 dislocates and degrades claudin-1 and claudin-2 at tight junctions of the intestinal epithelium. Front. Cell. Infect. Microbiol..

[55] Cornick S., Moreau F., Chadee K. (2016). *Entamoeba histolytica* Cysteine Proteinase 5 Evokes Mucin Exocytosis from Colonic Goblet Cells via αvβ3 Integrin. PLoS Pathog..

[56] García-Rivera G., Rodriguez M.A., Ocadiz R., Martinez-Lopez M.C., Arroyo R., Gonzalez-Robles A., Orozco E. (1999). *Entamoeba histolytica*: A novel cysteine protease and an adhesin form the 112 kDa surface protein. Mol. Microbiol..

[57] Montaño S., Orozco E., Correa-Basurto J., Bello M., Chávez-Munguía B., Betanzos A. (2017). Heterodimerization of the *Entamoeba histolytica* EhCPADH virulence complex through molecular dynamics and protein–protein docking. J. Biomol. Struct. Dyn..

[58] Ocádiz R., Orozco E., Carrillo E., Quintas L.I., Ortega-Lopez J., Garcia-Perez R.M., Sanchez T., Castillo-Juarez B.A., Garcia-Rivera G., Rodriguez M.A. (2005). EhCP112 is an Entamoeba histolytica secreted cysteine protease that may be involved in the parasite-virulence. Cell Microbiol..

[59] Bolaños J., Betanzos A., Javier-Reyna R., García- Rivera G., Huerta M., Pais-Morales J., González-Robles A., Rodríguez M.A., Schnoor M., Orozco E. (2016). EhNPC1 and EhNPC2 Proteins Participate in Trafficking of Exogenous Cholesterol in Entamoeba histolytica Trophozoites: Relevance for Phagocytosis. PLoS Pathog..

[60] Quintas-Granados L.I., Orozco E., Brieba L.G., Arroyo R., Ortega-Lopez J. (2009). Purification, refolding and autoactivation of the recombinant cysteine proteinase EhCP112 from *Entamoeba histolytica*. Protein Expr. Purif..

[61] Jacobs T., Bruchhaus I., Dandekar T., Tannich E., Leippe M. (1998). Isolation and molecular characterization of a surface-bound proteinase of Entamoeba histolytica. Mol. Microbiol..

[62] Moncada D., Keller K., Ankri S., Mirelman D., Chadee K. (2006). Antisense Inhibition of Entamoeba histolytica Cysteine Proteases Inhibits Colonic Mucus Degradation. Gastroenterology.

[63] Aguirre García M., Gutiérrez-Kobeh L., López Vancell R. (2015). Entamoeba histolytica: adhesins and lectins in the trophozoite surface. Molecules.

[64] Rosales-Encina J.L., Meza I., Lopez-De-Leon A., Talamas-Rohana P., Rojkind M. (1987). Isolation of a 220-kilodalton protein with lectin properties from a virulent strain of Entamoeba histolytica. J. Infect. Dis..

[65] Meza I., Cazares F., Rosales-Encina J.L., Talamas-Rohana P., Rojkind M. (1987). Use of antibodies to characterize a 220-kilodalton surface protein from Entamoeba histolytica. J. Infect. Dis..

[66] Seigneur M., Mounier J., Prevost M.C., Guillén N. (2005). A lysine- and glutamic acid-rich protein, KERP1, from Entamoeba histolytica binds to human enterocytes. Cell. Microbiol..

[67] Pineda E., Encalada R., Rodríguez-Zavala J.S., Olivos-García A., Moreno-Sánchez R., Saavedra E. (2010). Pyruvate:ferredoxin oxidoreductase and bifunctional aldehyde–alcohol dehydrogenase are essential for energy metabolism under oxidative stress in *Entamoeba histolytica*. FEBS J..

[68] Baxt L.A., Rastew E., Bracha R., Mirelman D., Singh U. (2010). Downregulation of an Entamoeba histolytica rhomboid protease reveals roles in regulating parasite adhesion and phagocytosis. Eukaryot. Cell.

[69] Bañuelos C., García-Rivera G., López-Reyes I., Mendoza L., González-Robles A., Herranz S., Vincent O., Orozco E. (2012). EhADH112 Is a Bro1 domain-containing protein involved in the entamoeba histolytica multivesicular bodies pathway. J. Biomed. Biotechnol..

[70] Betanzos A., Zanatta D., Bañuelos C., Hernández-Nava E., Cuellar P., Orozco E. (2018). Epithelial Cells Expressing EhADH, An Entamoeba histolytica Adhesin, Exhibit Increased Tight Junction Proteins. Front. Cell. Infect. Microbiol..

[71] Ocádiz-Ruiz R., Fonseca W., Linford A.S., Yoshino T.P., Orozco E., Rodríguez M.A. (2016). The knockdown of each component of the cysteine proteinase-adhesin complex of *Entamoeba histolytica* (EhCPADH) affects the expression of the other complex element as well as the in vitro and in vivo virulence. Parasitology.

[72] Hernández-Nava E., Cuellar P., Nava P., Chávez-Munguía B., Schnoor M., Orozco E., Betanzos A. (2017). Adherens junctions and desmosomes are damaged by *Entamoeba histolytica*: Participation of EhCPADH complex and EhCP112 protease. Cell. Microbiol..

[73] Vines R.R., Ramakrishnan G., Rogers J.B., Lockhart L.A., Mann B.J., Petri W.A. (1998). Regulation of adherence and virulence by the *Entamoeba histolytica* lectin cytoplasmic domain, which contains a β2 integrin motif. Mol. Biol. Cell.

[74] Alattia J.R., Kurokawa H., Ikura M. (1999). Structural view of cadherin-mediated cell-cell adhesion. Cell. Mol. Life Sci..

[75] Ravdin J.I., Murphy C.F., Salata R.A., Guerrant R.L., Hewlett E.L. (1985). *N*-Acetyl-d-galactosamine-inhibitable adherence lectin of *Entamoeba histolytica*. I. Partial purification and relation to amoebic virulence in vitro. J. Infect. Dis..

[76] Mittal K., Welter B.H., Temesvari L.A. (2008). *Entamoeba histolytica*: Lipid rafts are involved in adhesion of trophozoites to host extracellular matrix components. Exp. Parasitol..

[77] Naiyer S., Kaur D., Ahamad J., Singh S.S., Singh Y.P., Thakur V., Bhattacharya A., Bhattacharya S. (2019). Transcriptomic analysis reveals novel downstream regulatory motifs and highly transcribed virulence factor genes of *Entamoeba histolytica*. BMC Genom..

[78] Baxt L.A., Baker R.P., Singh U., Urban S. (2008). An *Entamoeba histolytica* rhomboid protease with atypical specificity cleaves a surface lectin involved in phagocytosis and immune evasion. Genes Dev..

[79] Perdomo D., Baron B., Rojo-Domínguez A., Raynal B., England P., Guillén N. (2013). The α-helical regions of KERP1 are important in *Entamoeba histolytica* adherence to human cells. Sci. Rep..

[80] Rodríguez M.A., García-Pérez R.M., Mendoza L., Sánchez T., Guillen N., Orozco E. (1998). The pyruvate:ferredoxin oxidoreductase enzyme is located in the plasma membrane and in a cytoplasmic structure inEntamoeba. Microb. Pathog..

[81] Leroy A., Lauwaet T., De Bruyne G., Cornelissen M., Mareel M. (2000). *Entamoeba histolytica* disturbs the tight junction complex in human enteric T84 cell layers. FASEB J..

[82] Betanzos A., Javier-Reyna R., García-Rivera G., Bañuelos C., González-Mariscal L., Schnoor M., Orozco E. (2013). The EhCPADH112 Complex of Entamoeba histolytica Interacts with Tight Junction Proteins Occludin and Claudin-1 to Produce Epithelial Damage. PLoS ONE.

[83] Cornick S., Chadee K. (2017). *Entamoeba histolytica*: Host parasite interactions at the colonic epithelium. Tissue Barriers.

[84] He C., Nora G.P., Schneider E.L., Kerr I.D., Hansell E., Hirata K., Gonzalez D., Sajid M., Boyd S.E., Hruz P. (2010). A novel *Entamoeba histolytica* cysteine proteinase, EhCP4, is key for invasive amebiasis and a therapeutic target. J. Biol. Chem..

[85] Lauwaet T., José Oliveira M., Callewaert B., De Bruyne G., Mareel M., Leroy A. (2004). Proteinase inhibitors TPCK and TLCK prevent *Entamoeba histolytica* induced disturbance of tight junctions and microvilli in enteric cell layers in vitro. Int. J. Parasitol..

[86] Lejeune M., Moreau F., Chadee K. (2011). Prostaglandin E2 produced by *Entamoeba histolytica* signals via EP4 receptor and alters claudin-4 to increase ion permeability of tight junctions. Am. J. Pathol..

[87] Goplen M., Lejeune M., Cornick S., Moreau F., Chadee K. (2013). *Entamoeba histolytica* contains an occludin-like protein that can alter colonic epithelial barrier function. PLoS One.

[88] Furuse M., Furuse K., Sasaki H., Tsukita S. (2001). Conversion of zonulae occludentes from tight to leaky strand type by introducing claudin-2 into Madin-Darby canine kidney I cells. J. Cell Biol..

[89] Lauwaet T., Oliveira M.J., Callewaert B., De Bruyne G., Saelens X., Ankri S., Vandenabeele P., Mirelman D., Mareel M., Leroy A. (2003). Proteolysis of enteric cell villin by *Entamoeba histolytica* cysteine proteinases. J. Biol. Chem..

[90] Pertuz Belloso S., Ostoa Saloma P., Benitez I., Soldevila G., Olivos A., García-Zepeda E. (2004). *Entamoeba histolytica* cysteine protease 2 (EhCP2) modulates leucocyte migration by proteolytic cleavage of chemokines. Parasite Immunol..

[91] Gilchrist C.A., Houpt E., Trapaidze N., Fei Z., Crasta O., Asgharpour A., Evans C., Martino-Catt S., Baba D.J., Stroup S. (2006). Impact of intestinal colonization and invasion on the *Entamoeba histolytica* transcriptome. Mol. Biochem. Parasitol..

[92] Zhang Z., Wang L., Seydel K.B., Li E., Ankri S., Mirelman D., Stanley S.L. (2000). Entamoeba histolytica cysteine proteinases with interleukin-1 beta converting enzyme (ICE) activity cause intestinal inflammation and tissue damage in amoebiasis. Mol. Microbiol..

[93] Nusrat A., Brown G.T., Tom J., Drake A., Bui T.T.T., Quan C., Mrsny R.J. (2005). Multiple protein interactions involving proposed extracellular loop domains of the tight junction protein occludin. Mol. Biol. Cell.

[94] Sateriale A., Huston C.D. (2011). A Sequential Model of Host Cell Killing and Phagocytosis by *Entamoeba histolytica*. J. Parasitol. Res..

[95] Ralston K.S., Petri W. (2011). The ways of a killer: How does *Entamoeba histolytica* elicit host cell death?. Essays Biochem..

[96] Leippe M., Herbst R. (2004). Ancient weapons for attack and defense: the pore-forming polypeptides of pathogenic enteric and free-living amoeboid protozoa. J. Eukaryot. Microbiol..

[97] Serrano-Luna J., Piña-Vázquez C., Reyes-López M., Ortiz-Estrada G., De La Garza M. (2013). Proteases from *Entamoeba* spp. and Pathogenic Free-Living Amoebae as Virulence Factors. J. Trop. Med..

[98] Ocádiz-Ruiz R., Fonseca W., Martínez M.B., Ocádiz-Quintanar R., Orozco E., Rodríguez M.A. (2013). Effect of the silencing of the Ehcp112 gene on the in vitro virulence of Entamoeba histolytica. Parasit. Vectors.

[99] Martinez M.B., Rodriguez M.A., Garcia-Rivera G., Sanchez T., Hernandez-Pando R., Aguilar D., Orozco E. (2009). A pcDNA-Ehcpadh vaccine against *Entamoeba histolytica* elicits a protective Th1-like response in hamster liver. Vaccine.

[100] Martinez-Lopez C., Orozco E., Sanchez T., Garcia-Perez R.M., Hernandez-Hernandez F., Rodriguez M.A. (2004). The EhADH112 recombinant polypeptide inhibits cell destruction and liver abscess formation by *Entamoeba histolytica* trophozoites. Cell Microbiol..

[101] Meléndez-López S.G., Herdman S., Hirata K., Choi M.H., Choe Y., Craik C., Caffrey C.R., Hansell E., Chávez-Munguía B., Yen T.C. (2007). Use of recombinant *Entamoeba histolytica* cysteine proteinase 1 to identify a potent inhibitor of amebic invasion in a human colonic model. Eukaryot. Cell.

[102] Irmer H., Tillack M., Biller L., Handal G., Leippe M., Roeder T., Tannich E., Bruchhaus I. (2009). Major cysteine peptidases of *Entamoeba histolytica* are required for aggregation and digestion of erythrocytes but are dispensable for phagocytosis and cytopathogenicity. Mol. Microbiol..

[103] Thibeaux R., Ave P., Bernier M., Morcelet M., Frileux P., Guillen N., Labruyere E., Avé P., Bernier M., Morcelet M. (2014). The parasite *Entamoeba histolytica* exploits the activities of human matrix metalloproteinases to invade colonic tissue. Nat. Commun..

[104] Leippe M. (1997). Amoebapores. Parasitol. Today.

[105] Zhang X., Zhang Z., Alexander D., Bracha R., Mirelman D., Stanley S.L. (2004). Expression of amoebapores is required for full expression of *Entamoeba histolytica* virulence in amebic liver abscess but is not necessary for the induction of inflammation or tissue damage in amebic colitis. Infect. Immun..

[106] Andra J., Berninghausen O., Leippe M. (1997). Potency of amoebapores compared to that of other membrane-permeating peptides. Arch. Med. Res..

[107] Sim S., Park S.-J., Yong T.-S., Im K.-I., Shin M.H. (2007). Involvement of β2-integrin in ROS-mediated neutrophil apoptosis induced by *Entamoeba histolytica*. Microbes Infect..

[108] Rodríguez M.A., Orozco E. (1986). Isolation and characterization of phagocytosis- and virulence-deficient mutants of *Entamoeba histolytica*. J. Infect. Dis..

[109] Orozco E., Guarneros G., Martinez-Palomo A., Sanchez T. (1983). *Entamoeba histolytica*. Phagocytosis as a virulence factor. J. Exp. Med..

[110] Huston C.D., Boettner D.R., Miller-Sims V., Petri W.A. (2003). Apoptotic Killing and Phagocytosis of Host Cells by the Parasite *Entamoeba histolytica*. Infect. Immun..

[111] Babuta M., Mansuri M.S., Bhattacharya S., Bhattacharya A. (2015). The Entamoeba histolytica, Arp2/3 Complex Is Recruited to Phagocytic Cups through an Atypical Kinase EhAK1. PLoS Pathog..

[112] Marion S., Laurent C., Guillen N. (2005). Signalization and cytoskeleton activity through myosin IB during the early steps of phagocytosis in *Entamoeba histolytica*: A proteomic approach. Cell Microbiol.

[113] Bhattacharya A., Padhan N., Jain R., Bhattacharya S. (2006). Calcium-Binding Proteins of *Entamoeba histolytica*. Arch. Med. Res..

[114] Nakada-Tsukui K., Saito-Nakano Y., Husain A., Nozaki T. (2010). Conservation and function of Rab small GTPases in *Entamoeba*: Annotation of E. invadens Rab and its use for the understanding of *Entamoeba* biology. Exp. Parasitol..

[115] Avalos-Padilla Y., Knorr R.L., Javier-Reyna R., García-Rivera G., Lipowsky R., Dimova R., Orozco E. (2018). The Conserved ESCRT-III Machinery Participates in the Phagocytosis of *Entamoeba histolytica*. Front. Cell. Infect. Microbiol..

[116] Vats D., Vishwakarma R.A., Bhattacharya S., Bhattacharya A. (2005). Reduction of cell surface glycosylphosphatidylinositol conjugates in *Entamoeba histolytica* by antisense blocking of *E. histolytica* GlcNAc-phosphatidylinositol deacetylase expression: effect on cell proliferation, endocytosis, and adhesion to target cells. Infect. Immun..

[117] Nakada-Tsukui K., Okada H., Mitra B.N., Nozaki T. (2009). Phosphatidylinositol-phosphates mediate cytoskeletal reorganization during phagocytosis via a unique modular protein consisting of RhoGEF/DH and FYVE domains in the parasitic protozoon *Entamoeba histolytica*. Cell. Microbiol..

[118] Avalos-Padilla Y., Betanzos A., Javier-Reyna R., Garcia-Rivera G., Chavez-Munguia B., Lagunes-Guillen A., Ortega J., Orozco E., García-Rivera G., Chávez-Munguía B. (2015). EhVps32 Is a Vacuole-Associated Protein Involved in Pinocytosis and Phagocytosis of Entamoeaba histolytica. PLoS Pathog.

[119] Katz U., Ankri S., Stolarsky T., Nuchamowitz Y., Mirelman D. (2002). *Entamoeba histolytica* expressing a dominant negative N-truncated light subunit of its gal-lectin are less virulent. Mol. Biol. Cell.

[120] Buss S.N., Hamano S., Vidrich A., Evans C., Zhang Y., Crasta O.R., Sobral B.W., Gilchrist C.A., Petri W.A. (2010). Members of the *Entamoeba histolytica* transmembrane kinase family play non-redundant roles in growth and phagocytosis. Int. J. Parasitol..

[121] Singh S.S., Naiyer S., Bharadwaj R., Kumar A., Singh Y.P., Ray A.K., Subbarao N., Bhattacharya A., Bhattacharya S. (2018). Stress-induced nuclear depletion of *Entamoeba histolytica* 3′-5′ exoribonuclease EhRrp6 and its role in growth and erythrophagocytosis. J. Biol. Chem..

[122] Teixeira J.E., Sateriale A., Bessoff K.E., Huston C.D. (2012). Control of *Entamoeba histolytica* adherence involves metallosurface protease 1, an M8 family surface metalloprotease with homology to leishmanolysin. Infect. Immun..

[123] Uribe R., Almaraz-Barrera M.J., Robles-Flores M., Mendoza-Hernández G., González-Robles A., Hernández-Rivas R., Guillen N., Vargas M. (2012). A functional study of nucleocytoplasmic transport signals of the EhNCABP166 protein from *Entamoeba histolytica*. Parasitology.

[124] Babuta M., Kumar S., Gourinath S., Bhattacharya S., Bhattacharya A. (2018). Calcium-binding protein EhCaBP3 is recruited to the phagocytic complex of *Entamoeba histolytica* by interacting with Arp2/3 complex subunit 2. Cell. Microbiol..

[125] Verma K., Srivastava V.K., Datta S. (2018). Rab GTPases take centre stage in understanding *Entamoeba histolytica* biology. Small GTPases.

[126] Welter B.H., Temesvari L.A. (2009). Overexpression of a mutant form of EhRabA, a unique Rab GTPase of *Entamoeba histolytica*, alters endoplasmic reticulum morphology and localization of the Gal/GalNAc adherence lectin. Eukaryot. Cell.

[127] Hernandes-Alejandro M., Calixto-Gálvez M., López-Reyes I., Salas-Casas A., Cázares-Ápatiga J., Orozco E., Rodríguez M.A. (2013). The small GTPase EhRabB of *Entamoeba histolytica* is differentially expressed during phagocytosis. Parasitol. Res..

[128] Saito-Nakano Y., Yasuda T., Nakada-Tsukui K., Leippe M., Nozaki T. (2004). Rab5-associated vacuoles play a unique role in phagocytosis of the enteric protozoan parasite *Entamoeba histolytica*. J. Biol. Chem..

[129] Saito-Nakano Y., Mitra B.N., Nakada-Tsukui K., Sato D., Nozaki T. (2007). Two Rab7 isotypes, EhRab7A and EhRab7B, play distinct roles in biogenesis of lysosomes and phagosomes in the enteric protozoan parasite *Entamoeba histolytica*. Cell. Microbiol..

[130] Hanadate Y., Saito-Nakano Y., Nakada-Tsukui K., Nozaki T. (2016). Endoplasmic reticulum-resident Rab8A GTPase is involved in phagocytosis in the protozoan parasite *Entamoeba histolytica*. Cell. Microbiol..

[131] Mitra B.N., Saito-Nakano Y., Nakada-Tsukui K., Sato D., Nozaki T. (2007). Rab11B small GTPase regulates secretion of cysteine proteases in the enteric protozoan parasite *Entamoeba histolytica*. Cell. Microbiol..

[132] Verma K., Datta S. (2017). Heavy subunit of cell surface Gal/GalNAc lectin (Hgl) undergoes degradation via endo-lysosomal compartments in Entamoeba histolytica. Small GTPases.

[133] Ghosh S.K., Samuelson J. (1997). Involvement of p21racA, phosphoinositide 3-kinase, and vacuolar ATPase in phagocytosis of bacteria and erythrocytes by *Entamoeba histolytica*: Suggestive evidence for coincidental evolution of amebic invasiveness. Infect. Immun..

[134] Bharadwaj R., Sharma S., Janhawi, Arya R., Bhattacharya S., Bhattacharya A. (2018). EhRho1 regulates phagocytosis by modulating actin dynamics through EhFormin1 and EhProfilin1 in *Entamoeba histolytica*. Cell. Microbiol..

[135] López-Reyes I., Bañuelos C., Betanzos A., Orozco E. (2011). A bioinformatical approach to study the endosomal sorting complex required for transport (ESCRT) machinery in protozoan parasites: the Entamoeba histolytica case. Bioinform. Trends Methodol..

[136] López-Reyes I., García-Rivera G., Bauelos C., Herranz S., Vincent O., Lpez-Camarillo C., Marchat L.A., Orozco E., López-Reyes I., García-Rivera G. (2010). Detection of the Endosomal Sorting Complex Required for Transport in *Entamoeba histolytica* and Characterization of the EhVps4 Protein. J Biomed Biotechnol.

[137] Castellanos-Castro S., Cerda-García-Rojas C.M., Javier-Reyna R., Pais-Morales J., Chávez-Munguía B., Orozco E. (2016). Identification of the phospholipid lysobisphosphatidic acid in the protozoan *Entamoeba histolytica*: An active molecule in endocytosis. Biochem. Biophys. Reports.

[138] Castellanos-Castro S., Montaño S., Orozco E. (2016). Data on docking and dynamics simulation of *Entamoeba histolytica* EhADH (an ALIX protein) and lysobisphosphatidic acid. Data Br..

[139] Gilmartin A.A., Petri W.A. (2017). Exploring the mechanism of amebic trogocytosis: the role of amebic lysosomes. Microb. Cell.

[140] Ralston K.S., Solga M.D., Mackey-Lawrence N.M., Somlata, Bhattacharya A., Petri W.A. (2014). Trogocytosis by *Entamoeba histolytica* contributes to cell killing and tissue invasion. Nature.

[141] Somlata, Nakada-Tsukui K., Nozaki T. (2017). AGC family kinase 1 participates in trogocytosis but not in phagocytosis in *Entamoeba histolytica*. Nat. Commun..

[142] Guerrant R.L., Brush J., Ravdin J.I., Sullivan J.A., Mandell G.L. (1981). Interaction between *Entamoeba histolytica* and human polymorphonuclear neutrophils. J. Infect. Dis..

[143] Denis M., Chadee K. (1989). Human Neutrophils Activated by Interferon-γ and Tumour Necrosis Factor-α Kill *Entamoeba histolytica* Trophozoites In Vitro. J. Leukoc. Biol..

[144] Kammanadiminti S.J., Mann B.J., Dutil L., Chadee K. (2003). Regulation of Toll-like receptor-2 expression by the Gal-lectin of *Entamoeba histolytica*. FASEB J..

[145] Lin J.Y., Seguin R., Keller K., Chadee K. (1994). Tumor necrosis factor α augments nitric oxide-dependent macrophage cytotoxicity against *Entamoeba histolytica* by enhanced expression of the nitric oxide synthase gene. Infect. Immun..

[146] Haque R., Mondal D., Shu J., Roy S., Kabir M., Davis A.N., Duggal P., Petri W.A. (2007). Correlation of interferon-γ production by peripheral blood mononuclear cells with childhood malnutrition and susceptibility to amebiasis. Am. J. Trop. Med. Hyg..

[147] Dey I., Keller K., Belley A., Chadee K. (2003). Identification and characterization of a cyclooxygenase-like enzyme from *Entamoeba histolytica*. Proc. Natl. Acad. Sci. USA.

[148] Lee Y.A., Nam Y.H., Min A., Kim K.A., Nozaki T., Saito-Nakano Y., Mirelman D., Shin M.H. (2014). *Entamoeba histolytica*-secreted cysteine proteases induce IL-8 production in human mast cells via a PAR2-independent mechanism. Parasite.

[149] Mortimer L., Moreau F., Cornick S., Chadee K. (2013). Gal-lectin-dependent contact activates the inflammasome by invasive *Entamoeba histolytica*. Mucosal Immunol..

[150] Mortimer L., Moreau F., Cornick S., Chadee K. (2015). The NLRP3 Inflammasome Is a Pathogen Sensor for Invasive *Entamoeba histolytica* via Activation of α5β1 Integrin at the Macrophage-Amebae Intercellular Junction. PLoS Pathog..

[151] Cobo E.R., He C., Hirata K., Hwang G., Tran U., Eckmann L., Gallo R.L., Reed S.L. (2012). *Entamoeba histolytica* induces intestinal cathelicidins but is resistant to cathelicidin-mediated killing. Infect. Immun..

[152] Davis P.H., Zhang X., Guo J., Townsend R.R., Stanley S.L. (2006). Comparative proteomic analysis of two *Entamoeba histolytica* strains with different virulence phenotypes identifies peroxiredoxin as an important component of amoebic virulence. Mol. Microbiol..

[153] Jeelani G., Nozaki T. (2016). Entamoeba thiol-based redox metabolism: A potential target for drug development. Mol. Biochem. Parasitol..

[154] Sim S., Yong T.-S., Park S.-J., Im K., Kong Y., Ryu J.-S., Min D.-Y., Shin M.H. (2005). NADPH Oxidase-Derived Reactive Oxygen Species-Mediated Activation of ERK1/2 Is Required for Apoptosis of Human Neutrophils Induced by *Entamoeba histolytica*. J. Immunol..

[155] Lin J.Y., Keller K., Chadee K. (1993). Entamoeba histolytica proteins modulate the respiratory burst potential by murine macrophages. Immunology.

[156] Wang W., Keller K., Chadee K. (1994). *Entamoeba histolytica* modulates the nitric oxide synthase gene and nitric oxide production by macrophages for cytotoxicity against amoebae and tumour cells. Immunology.

[157] Braga L.L., Ninomiya H., McCoy J.J., Eacker S., Wiedmer T., Pham C., Wood S., Sims P.J., Petri W.A. (1992). Inhibition of the complement membrane attack complex by the galactose-specific adhesion of *Entamoeba histolytica*. J. Clin. Invest..

[158] Reed S.L., Ember J.A., Herdman D.S., DiScipio R.G., Hugli T.E., Gigli I. (1995). The extracellular neutral cysteine proteinase of *Entamoeba histolytica* degrades anaphylatoxins C3a and C5a. J. Immunol..

[159] Zambrano-Villa S., Rosales-Borjas D., Carrero J.C., Ortiz-Ortiz L. (2002). How protozoan parasites evade the immune response. Trends Parasitol..

[160] Que X., Reed S.L. (1997). The role of extracellular cysteine proteinases in pathogenesis of *Entamoeba histolytica* invasion. Parasitol. Today.

[161] Morton E.R., Lynch J., Froment A., Lafosse S., Heyer E., Przeworski M., Blekhman R., Ségurel L. (2015). Variation in Rural African Gut Microbiota Is Strongly Correlated with Colonization by *Entamoeba* and Subsistence. PLoS Genet..

[162] Burgess S.L., Petri W.A. (2016). The Intestinal Bacterial Microbiome and *E. histolytica* Infection. Curr. Trop. Med. reports.

[163] Bracha R., Mirelman D. (1984). Virulence of *Entamoeba histolytica* trophozoites. Effects of bacteria, microaerobic conditions, and metronidazole. J. Exp. Med..

[164] Phillips B.P., Gorstein F. (1966). Effects of Different Species of Bacteria on the Pathology of Enteric Amebiasis in Monocontaminated Guinea Pigs. Am. J. Trop. Med. Hyg..

[165] Verma A.K., Verma R., Ahuja V., Paul J. (2012). Real-time analysis of gut flora in *Entamoeba histolytica* infected patients of Northern India. BMC Microbiol..

[166] Gilchrist C.A., Petri S.E., Schneider B.N., Reichman D.J., Jiang N., Begum S., Watanabe K., Jansen C.S., Elliott K.P., Burgess S.L. (2016). Role of the Gut Microbiota of Children in Diarrhea Due to the Protozoan Parasite *Entamoeba histolytica*. J. Infect. Dis..

[167] Scher J.U., Sczesnak A., Longman R.S., Segata N., Ubeda C., Bielski C., Rostron T., Cerundolo V., Pamer E.G., Abramson S.B. (2013). Expansion of intestinal Prevotella copri correlates with enhanced susceptibility to arthritis. eLife.

[168] Morgado P., Manna D., Singh U. (2016). Recent advances in *Entamoeba* biology: RNA interference, drug discovery, and gut microbiome. F1000Research.

[169] Piña-Vázquez C., Reyes-López M., Ortíz-Estrada G., de la Garza M., Serrano-Luna J. (2012). Host-parasite interaction: parasite-derived and -induced proteases that degrade human extracellular matrix. J. Parasitol. Res..

[170] Labruyère E., Guillén N. (2006). Host tissue invasion by *Entamoeba histolytica* is powered by motility and phagocytosis. Arch. Med. Res..

[171] Rojas-Hernández S., Jarillo-Luna A., Rodríguez-Monroy M., Moreno-Fierros L., Campos-Rodríguez R. (2004). Immunohistochemical characterization of the initial stages of *Naegleria fowleri* meningoencephalitis in mice. Parasitol. Res..

[172] Visvesvara G.S., Callaway C.S. (1974). Light and Electron Microsopic Observations on the Pathogenesis of *Naegleria fowleri* in Mouse Brain and Tissue Culture. J. Protozool..

[173] Jarolim L.K., McCosh K.J., Howard J.M. (2002). The role of blood vessels and lungs in the dissemination of *Naegleria fowleri* following intranasal inoculation in mice. Folia Parasitol..

[174] Cervantes-Sandoval I., Jesús Serrano-Luna J., Pacheco-Yépez J., Silva-Olivares A., Tsutsumi V., Shibayama M. (2010). Differences between *Naegleria fowleri* and *Naegleria gruberi* in expression of mannose and fucose glycoconjugates. Parasitol. Res..

[175] Pervin N., Sundareshan V. (2018). Naegleria.

[176] Cervantes-Sandoval I., Serrano-Luna J.d.J., García-Latorre E., Tsutsumi V., Shibayama M. (2008). Mucins in the host defence against *Naegleria fowleri* and mucinolytic activity as a possible means of evasion. Microbiology.

[177] Marciano-Cabral F.M., Patterson M., John D.T., Bradley S.Q. (1982). Cytopathogenicity of *Naegleria fowleri* and *Naegleria gruberi* for Established Mammalian Cell Cultures. J. Parasitol..

[178] Marciano-Cabral F. (1988). Biology of *Naegleria* spp.. Microbiol. Rev..

[179] Carrasco-Yepez M., Campos-Rodriguez R., Godinez-Victoria M., Rodriguez-Monroy M.A., Jarillo-Luna A., Bonilla-Lemus P., De Oca A.C.-M., Rojas-Hernandez S. (2013). *Naegleria fowleri* glycoconjugates with residues of α-d-mannose are involved in adherence of trophozoites to mouse nasal mucosa. Parasitol. Res..

[180] Han K.L., Lee H.J., Shin M.H., Shin H.J., Im K.I., Park S.J. (2004). The involvement of an integrin-like protein and protein kinase C in amoebic adhesion to fibronectin and amoebic cytotoxicity. Parasitol. Res..

[181] Shibayama M., Serrano-Luna J.D.J., Rojas-Hernández S., Campos-Rodríguez R., Tsutsumi V. (2003). Interaction of secretory immunoglobulin A antibodies with *Naegleria fowleri* trophozoites and collagen type I. Can. J. Microbiol..

[182] Jamerson M., da Rocha-Azevedo B., Cabral G.A., Marciano-Cabral F. (2012). Pathogenic *Naegleria fowleri* and non-pathogenic *Naegleria lovaniensis* exhibit differential adhesion to, and invasion of, extracellular matrix proteins. Microbiology.

[183] Cervantes-Sandoval I., Serrano-Luna J.d.J., García-Latorre E., Tsutsumi V., Shibayama M. (2008). Characterization of brain inflammation during primary amoebic meningoencephalitis. Parasitol. Int..

[184] Shibayama M., Martínez-Castillo M., Silva-Olivares A., Galindo-Gómez S., Navarro-García F., Escobar-Herrera J., Sabanero M., Tsutsumi V., Serrano-Luna J. (2013). Disruption of MDCK cell tight junctions by the free-living amoeba *Naegleria fowleri*. Microbiology.

[185] Coronado-Velázquez D., Betanzos A., Serrano-Luna J., Shibayama M. (2018). An In Vitro Model of the Blood-Brain Barrier: *Naegleria fowleri* Affects the Tight Junction Proteins and Activates the Microvascular Endothelial Cells. J. Eukaryot. Microbiol..

[186] Aldape K., Huizinga H., Bouvier J., Mckerrow J. (1994). *Naegleria fowleri*: Characterization of a Secreted Histolytic Cysteine Protease. Exp. Parasitol..

[187] Ferrante A., Bates E.J. (1988). Elastase in the pathogenic free-living amoebae *Naegleria* and *Acanthamoeba* spp.. Infect. Immun..

[188] Fulford D.E., Marciano-Cabral F. (1986). Cytolytic Activity of *Naegleria fowleri* Cell-free Extract. J. Protozool..

[189] Eisen D., Franson R.C. (1987). Acid-active neuraminidases in the growht media from cultures of pathogenic *Naegleria fowleri* and in sonicates of rabbit alveolar macrophages. Biochim. Biophys. Acta - Gen. Subj..

[190] Herbst R., Ott C., Jacobs T., Marti T., Marciano-Cabral F., Leippe M. (2002). Pore-forming Polypeptides of the Pathogenic Protozoon *Naegleria fowleri*. J. Biol. Chem..

[191] Hysmith R.M., Franson R.C. (1982). Elevated levels of cellular and extracellular phospholipases from pathogenic *Naegleria fowleri*. Biochim. Biophys. Acta - Lipids Lipid Metab..

[192] Zyserman I., Mondal D., Sarabia F., McKerrow J.H., Roush W.R., Debnath A. (2018). Identification of cysteine protease inhibitors as new drug leads against *Naegleria fowleri*. Exp. Parasitol..

[193] Lee J., Kim J.-H., Sohn H.-J., Yang H.-J., Na B.-K., Chwae Y.-J., Park S., Kim K., Shin H.-J. (2014). Novel cathepsin B and cathepsin B-like cysteine protease of *Naegleria fowleri* excretory–secretory proteins and their biochemical properties. Parasitol. Res..

[194] Brown T. (1979). Observations by immunofluorescence microscopy and electron microscopy on the cytopathogenicity of *Naegleria fowleri* in mouse embryo-cell cultures. J. Med. Microbiol..

[195] Marciano-Cabral F., John D.T. (1983). Cytopathogenicity of *Naegleria fowleri* for rat neuroblastoma cell cultures: Scanning electron microscopy study. Infect. Immun..

[196] John D.T., Cole T.B., Bruner R.A. (1985). Amebostomes of *Naegleria fowleri*. J. Protozool..

[197] Kang S.-Y., Song K.-J., Jeong S.-R., Kim J.-H., Park S., Kim K., Kwon M.-H., Shin H.-J. (2005). Role of the Nfa1 protein in pathogenic *Naegleria fowleri* cocultured with CHO target cells. Clin. Diagn. Lab. Immunol..

[198] Walsh C.J. (2007). The role of actin, actomyosin and microtubules in defining cell shape during the differentiation of Naegleria amebae into flagellates. Eur. J. Cell Biol..

[199] Sohn H.-J., Kim J.-H., Shin M.-H., Song K.-J., Shin H.-J. (2010). The Nf-actin gene is an important factor for food-cup formation and cytotoxicity of pathogenic *Naegleria fowleri*. Parasitol. Res..

[200] John D.T. (1982). Primary Amebic Meningoencephalitis and the Biology of *Naegleria Fowleri*. Annu. Rev. Microbiol..

[201] Marciano-Cabral F., Cabral G.A. (2007). The immune response to *Naegleria fowleri* amebae and pathogenesis of infection. FEMS Immunol. Med. Microbiol..

[202] Ferrante A., Mocatta T.J. (1984). Human neutrophils require activation by mononuclear leucocyte conditioned medium to kill the pathogenic free-living amoeba, *Naegleria fowleri*. Clin. Exp. Immunol..

[203] Ferrante A., Carter R.F., Lopez A.F., Rowan-Kelly B., Hill N.L., Vadas M.A. (1988). Depression of immunity to *Naegleria fowleri* in mice by selective depletion of neutrophils with a monoclonal antibody. Infect. Immun..

[204] Kim J.-H., Song A.-R., Sohn H.-J., Lee J., Yoo J.-K., Kwon D., Shin H.-J. (2013). IL-1β and IL-6 activate inflammatory responses of astrocytes against *Naegleria fowleri* infection via the modulation of MAPKs and AP-1. Parasite Immunol..

[205] Cursons R.T., Brown T.J., Keys E.A., Moriarty K.M., Till D. (1980). Immunity to pathogenic free-living amoebae: role of humoral antibody. Infect. Immun..

[206] Rivera-Aguilar V., Hernández-Martínez D., Rojas-Hernández S., Oliver-Aguillón G., Tsutsumi V., Herrera-González N., Campos-Rodríguez R. (2000). Immunoblot analysis of IgA antibodies to *Naegleria fowleri* in human saliva and serum. Parasitol. Res..

[207] Rivera V., Rojas S., Oliver G., Tsutsumi V., Hernández D., Shibayama M., Serrano J., Campos R. (2001). IgA and IgM anti-*Naegleria fowleri* antibodies in human serum and saliva. Can. J. Microbiol..

[208] Reilly M.F., White K.L., Bradley S.G. (1983). Host resistance of mice to *Naegleria fowleri* infections. Infect. Immun..

[209] Chu D.-M., Woodward J., Fritzinger A., Marciano-Cabral F. (2002). Calcium-dependent protection from complement lysis in *Naegleria fowleri* amebae. Cell Calcium.

[210] Fritzinger A.E., Toney D.M., MacLean R.C., Marciano-Cabral F. (2006). Identification of a *Naegleria fowleri* membrane protein reactive with anti-human CD59 antibody. Infect. Immun..

[211] Fritzinger A.E., Marciano-Cabral F. (2004). Modulation of a “CD59-like” Protein in *Naegleria fowleri* Amebae by Bacteria1. J. Eukaryot. Microbiol..

[212] Toney D.M., Marciano-Cabral F. (1994). Membrane vesiculation of *Naegleria fowleri* amoebae as a mechanism for resisting complement damage. J. Immunol..

[213] Chu D.-M.T., Ferguson T.J., Marciano-Cabral F. (2000). Protein Kinase Activation and Protein Phosphorylation in *Naegleria fowleri* Amebae in Response to Normal Human Serum. J. Eukaryot. Microbiol..

[214] Ferrante A., Thong Y.H. (1979). Antibody induced capping and endocytosis of surface antigens in *Naegleria fowleri*. Int. J. Parasitol..

[215] Siddiqui R., Khan N.A. (2012). Biology and pathogenesis of *Acanthamoeba*. Parasites Vectors.

[216] Clarke D.W., Niederkorn J.Y. (2006). The pathophysiology of *Acanthamoeba keratitis*. Trends Parasitol..

[217] Pettit D.A.D., Williamson J., Cabral G.A., Marciano-Cabral F. (2006). In vitro Destruction of Nerve Cell Cultures by *Acanthamoeba* spp.: A Transmission and Scanning Electron Microscopy Study. J. Parasitol..

[218] Omaña-Molina M., Navarro-García F., González-Robles A., Serrano-Luna J.d.J., Campos-Rodríguez R., Martínez-Palomo A., Tsutsumi V., Shibayama M. (2004). Induction of morphological and electrophysiological changes in hamster cornea after in vitro interaction with trophozoites of *Acanthamoeba* spp.. Infect. Immun..

[219] Martinez A.J., Janitschke K. (1985). Acanthamoeba, an opportunistic microorganism: A review. Infection.

[220] Sissons J., Alsam S., Goldsworthy G., Lightfoot M., Jarroll E.L., Khan N.A. (2006). Identification and properties of proteases from an Acanthamoeba isolate capable of producing granulomatous encephalitis. BMC Microbiol..

[221] Martinez A.J., Visvesvara G.S. (1997). Free-living, Amphizoic and Opportunistic Amebas. Brain Pathol..

[222] Kennett M.J., Hook R.R., Franklin C.L., Riley L.K. (1999). *Acanthamoeba castellanii*: Characterization of an Adhesin Molecule. Exp. Parasitol..

[223] Garate M., Cubillos I., Marchant J., Panjwani N. (2005). Biochemical characterization and functional studies of Acanthamoeba mannose-binding protein. Infect. Immun..

[224] Ng S.-L., Nordin A., Abd Ghafar N., Suboh Y., Ab Rahim N., Chua K.-H. (2017). Acanthamoeba-mediated cytopathic effect correlates with MBP and AhLBP mRNA expression. Parasit. Vectors.

[225] Hurt M., Neelam S., Niederkorn J., Alizadeh H. (2003). Pathogenic *Acanthamoeba* spp secrete a mannose-induced cytolytic protein that correlates with the ability to cause disease. Infect. Immun..

[226] Garate M., Marchant J., Cubillos I., Cao Z., Khan N.A., Panjwani N. (2006). In Vitro Pathogenicity of Acanthamoeba Is Associated with the Expression of the Mannose-Binding Protein. Invest. Ophthalmol. Vis. Sci..

[227] Alizadeh H., Li H., Neelam S., Niederkorn J.Y. (2008). Modulation of corneal and stromal matrix metalloproteinase by the mannose-induced Acanthamoeba cytolytic protein. Exp. Eye Res..

[228] Hong Y.-C., Lee W.-M., Kong H.-H., Jeong H.-J., Chung D.-I. (2004). Molecular cloning and characterization of a cDNA encoding a laminin-binding protein (AhLBP) from *Acanthamoeba healyi*. Exp. Parasitol..

[229] Gordon V.R., Asem E.K., Vodkin M.H., McLaughlin G.L. (1993). Acanthamoeba binds to extracellular matrix proteins in vitro. Invest. Ophthalmol. Vis. Sci..

[230] Wang L., Asem E.K., McLaughlin G.L. (1994). Calcium enhances Acanthamoeba polyphaga binding to extracellular matrix proteins. Invest. Ophthalmol. Vis. Sci..

[231] Rocha-Azevedo B.D.A., Jamerson M., Cabral G.U.Y.A., Silva-Filho F.C., Marciano-Cabral F. (2009). Acanthamoeba Interaction with Extracellular Matrix Glycoproteins: Biological and Biochemical Characterization and Role in Cytotoxicity and Invasiveness. J. Eukaryot. Microbiol..

[232] Moore M.B., Ubelaker J.E., Martin J.H., Silvany R., Dougherty J.M., Meyer D.R., McCulley J.P. (1991). In vitro penetration of human corneal epithelium by Acanthamoeba castellanii: A scanning and transmission electron microscopy study. Cornea.

[233] Khan N.A., Siddiqui R. (2009). Acanthamoeba affects the integrity of human brain microvascular endothelial cells and degrades the tight junction proteins. Int. J. Parasitol..

[234] Flores-Maldonado C., González-Robles A., Salazar-Villatoro L., Omaña-Molina M., Gallardo J.M., González-Lázaro M., Hernández-Ramírez V.I., Talamás-Rohana P., Lorenzo-Morales J., Martínez-Palomo A. (2017). Acanthamoeba (T4) trophozoites cross the MDCK epithelium without cell damage but increase paracellular permeability and transepithelial resistance by modifying tight junction composition. Exp. Parasitol..

[235] González-Robles A., Omaña-Molina M., Salazar-Villatoro L., Flores-Maldonado C., Lorenzo-Morales J., Reyes-Batlle M., Arnalich-Montiel F., Martínez-Palomo A. (2017). *Acanthamoeba culbertsoni* isolated from a clinical case with intraocular dissemination: Structure and in vitro analysis of the interaction with hamster cornea and MDCK epithelial cell monolayers. Exp. Parasitol..

[236] Khan N.A., Jarroll E.L., Panjwani N., Cao Z., Paget T.A. (2000). Proteases as markers for differentiation of pathogenic and nonpathogenic species of Acanthamoeba. J. Clin. Microbiol..

[237] Kim H.-K., Ha Y.-R., Yu H.-S., Kong H.-H., Chung D.-I. (2003). Purification and characterization of a 33 kDa serine protease from *Acanthamoeba lugdunensis* KA/E2 isolated from a Korean keratitis patient. Korean J. Parasitol..

[238] Alizadeh H., Pidherney M.S., McCulley J.P., Niederkorn J.Y. (1994). Apoptosis as a mechanism of cytolysis of tumor cells by a pathogenic free-living amoeba. Infect. Immun..

[239] de Souza Carvalho F.R., Carrijo-Carvalho L.C., Chudzinski-Tavassi A.M., Foronda A.S., de Freitas D. (2011). Serine-like proteolytic enzymes correlated with differential pathogenicity in patients with acute Acanthamoeba keratitis. Clin. Microbiol. Infect..

[240] Hadas E., Mazur T. (1993). Proteolytic enzymes of pathogenic and non-pathogenic strains of *Acanthamoeba* sp.. Trop. Med. Parasitol..

[241] Leher H., Silvany R., Alizadeh H., Huang J., Niederkorn J.Y. (1998). Mannose induces the release of cytopathic factors from *Acanthamoeba castellanii*. Infect. Immun..

[242] Panjwani N. (2010). Pathogenesis of *Acanthamoeba keratitis*. Ocul. Surf..

[243] Leitsch D., Köhsler M., Marchetti-Deschmann M., Deutsch A., Allmaier G., Duchêne M., Walochnik J. (2010). Major role for cysteine proteases during the early phase of *Acanthamoeba castellanii* encystment. Eukaryot. Cell.

[244] Serrano-Luna J.d.J., Cervantes-Sandoval I., Calderón J., Navarro-García F., Tsutsumi V., Shibayama M. (2006). Protease activities of *Acanthamoeba polyphaga* and *Acanthamoeba castellanii*. Can. J. Microbiol..

[245] Tripathi T., Abdi M., Alizadeh H. (2014). Protease-activated receptor 2 (PAR2) is upregulated by Acanthamoeba plasminogen activator (aPA) and induces proinflammatory cytokine in human corneal epithelial cells. Invest. Ophthalmol. Vis. Sci..

[246] Hong Y., Kang J.-M., Joo S.-Y., Song S.-M., Lê H.G., Thái T.L., Lee J., Goo Y.-K., Chung D.-I., Sohn W.-M. (2018). Molecular and Biochemical Properties of a Cysteine Protease of *Acanthamoeba castellanii*. Korean J. Parasitol..

[247] Ramírez-Rico G., Martínez-Castillo M., de la Garza M., Shibayama M., Serrano-Luna J. (2015). *Acanthamoeba castellanii* Proteases are Capable of Degrading Iron-Binding Proteins as a Possible Mechanism of Pathogenicity. J. Eukaryot. Microbiol..

[248] Yun H.C., Kim K.Y., Park S.Y., Park S.K., Park H., Hwang U.W., Hong K.M., Ryu J.S., Min D.Y. (1999). Cloning of a Cysteine Proteinase Gene from *Acanthamoeba culbertsoni*. Mol. Cells.

[249] Hong Y.-C., Hwang M.-Y., Yun H.-C., Yu H.-S., Kong H.-H., Yong T.-S., Chung D.-I. (2002). Isolation and characterization of a cDNA encoding a mammalian cathepsin L-like cysteine proteinase from Acanthamoeba healyi. Korean J. Parasitol..

[250] Michalek M., Sönnichsen F.D., Wechselberger R., Dingley A.J., Hung C.-W., Kopp A., Wienk H., Simanski M., Herbst R., Lorenzen I. (2012). Structure and function of a unique pore-forming protein from a pathogenic acanthamoeba. Nat. Chem. Biol..

[251] Siddiqui R., Khan N.A. (2012). Acanthamoeba is an evolutionary ancestor of macrophages: A myth or reality?. Exp. Parasitol..

[252] Alsam S., Sissons J., Dudley R., Khan N.A. (2005). Mechanisms associated with *Acanthamoeba castellanii* (T4) phagocytosis. Parasitol. Res..

[253] Chusattayanond A.D., Boonsilp S., Kasisit J., Boonmee A., Warit S. (2010). Thai Acanthamoeba isolate (T4) induced apoptotic death in neuroblastoma cells via the Bax-mediated pathway. Parasitol. Int..

[254] Clarke D.W., Niederkorn J.Y. (2006). The immunobiology of Acanthamoeba keratitis. Microbes Infect..

[255] Alizadeh H., Tripathi T., Abdi M., Smith A.D. (2014). Pathogenic strains of Acanthamoeba are recognized by TLR4 and initiated inflammatory responses in the cornea. PLoS ONE.

[256] Hurt M., Apte S., Leher H., Howard K., Niederkorn J., Alizadeh H. (2001). Exacerbation of Acanthamoeba keratitis in animals treated with anti-macrophage inflammatory protein 2 or antineutrophil antibodies. Infect. Immun..

[257] Lorenzo-Morales J., Khan N.A., Walochnik J. (2015). An update on *Acanthamoeba* keratitis: diagnosis, pathogenesis and treatment. Parasite.

[258] Pumidonming W., Walochnik J., Dauber E., Petry F. (2011). Binding to complement factors and activation of the alternative pathway by *Acanthamoeba*. Immunobiology.

[259] Campos-Rodríguez R., Oliver-Aguillón G., Vega-Pérez L.M., Jarillo-Luna A., Hernández-Martínez D., Rojas-Hernández S., Rodríguez-Monroy M.A., Rivera-Aguilar V., González-Robles A. (2004). Human IgA inhibits adherence of *Acanthamoeba polyphaga* to epithelial cells and contact lenses. Can. J. Microbiol..

[260] Alizadeh H., Apte S., El-Agha M.S.H., Li L., Hurt M., Howard K., Cavanagh H.D., McCulley J.P., Niederkorn J.Y. (2001). Tear IgA and serum IgG antibodies against Acanthamoeba in patients with Acanthamoeba keratitis. Cornea.

[261] Younus H. (2018). Therapeutic potentials of superoxide dismutase. Int. J. Health Sci. (Qassim).

[262] Choi D.-H., Na B.-K., Seo M.-S., Song H.-R., Song C.-Y. (2000). Purification and characterization of iron superoxide dismutase and copper–zinc superoxide dismutase from *Acanthamoeba castellanii*. J. Parasitol..

[263] Kim J.-Y., Na B.-K., Song K.-J., Park M.-H., Park Y.-K., Kim T.-S. (2012). Functional expression and characterization of an iron-containing superoxide dismutase of *Acanthamoeba castellanii*. Parasitol. Res..

